# Design and synthesis of strong root gravitropism inhibitors with no concomitant growth inhibition

**DOI:** 10.1038/s41598-023-32063-z

**Published:** 2023-03-30

**Authors:** Takeshi Nishimura, Saki Makigawa, Jun Sun, Kozue Kodama, Hiromi Sugiyama, Kenji Matsumoto, Takayuki Iwata, Naoya Wasano, Arihiro Kano, Miyo Terao Morita, Yoshiharu Fujii, Mitsuru Shindo

**Affiliations:** 1 Institute for Materials Chemistry and Engineering, Kyushu University, Kasuga-koen, Kasuga, 816-8580 Japan; 2 Interdisciplinary Graduate School of Engineering Sciences, Kyushu University, Kasuga-koen, Kasuga, 816-8580 Japan; 3 International Environmental and Agricultural Sciences, Tokyo University of Agriculture and Technology, 3-5-8, Saiwai-cho, Fuchu, Tokyo 183-8509 Japan; 4 Division of Plant Environmental Responses, National Institute for Basic Biology, Nishigonaka 38, Myodaiji, Okazaki 444-8585 Japan; 5 Present Address: Department of Engineering, Graduate School of Science and Engineering, Kagoshima University, Kagoshima, Japan; 6 Present Address: Institute of Biological Control, Faculty of Agriculture, Kyushu University, Fukuoka, Japan

**Keywords:** Plant sciences, Chemistry

## Abstract

Herein, we describe a highly potent gravitropic bending inhibitor with no concomitant growth inhibition. Previously, we reported that (*2Z,4E*)-5-phenylpenta-2,4-dienoic acid (ku-76) selectively inhibits root gravitropic bending of lettuce radicles at 5 μM. Based on the structure–activity relationship study of ku-76 as a lead compound, we designed and synthesized various C4-substituted analogs of ku-76. Among the analogs, 4-phenylethynyl analog exhibited the highest potency for gravitropic bending inhibition, which was effective at only 0.01 μM. Remarkably, 4-phenylethynyl analog is much more potent than the known inhibitor, NPA. Substitution in the *para* position on the aromatic ring of 4-phenylethynyl group was tolerated without diminished activity. In addition, evaluation using *Arabidopsis* indicated that 4-phenylethynyl analog inhibits gravitropism by affecting auxin distribution in the root tips. Based on the effects on *Arabidopsis* phenotypes, 4-phenylethynyl analog may be a novel inhibitor that differs in action from the previously reported auxin transport inhibitors.

## Introduction

The development of curvature of plant organs in response to gravity is called gravitropism, which includes the downward growth of roots (positive gravitropism) and the upward growth of shoots (negative gravitropism). The gravitropism mechanism includes several sequential processes^1,2^, wherein auxins play crucial roles in signal transmission for asymmetric organ growth^3–5^.

**Figure 1:**
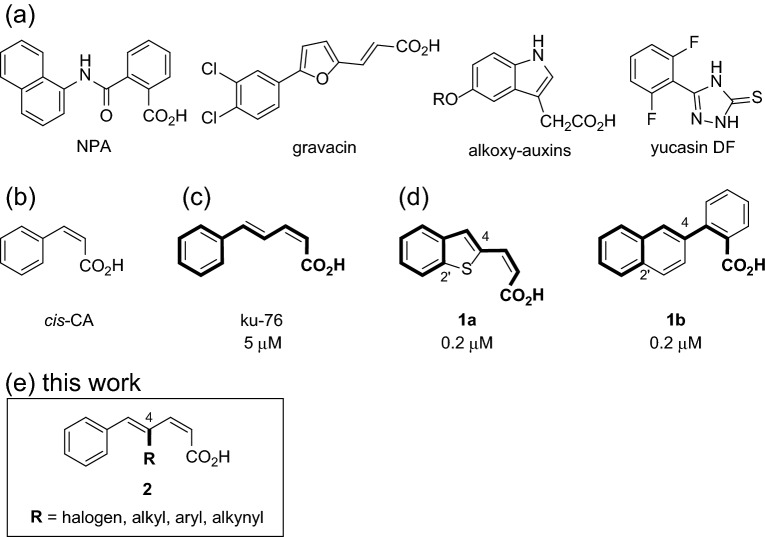
Structure of reported gravitropic inhibitors. (**a**) Non-selective gravitropic inhibitors of both elongation and gravitropic bending: NPA, gravacin, alkoxy-auxins, and yucasin DF (**b**) *cis*-CA. (**c**) ku-76. (**d**) More potent analogs, **1a** and **1b**, were reported previously. (**e**) C4-substituted ku-76 analogs that were synthesized in this study.

Gravitropic inhibitory chemical compounds contribute to the regulation of plant growth, leading to the development of novel weed suppressors and the elucidation of gravitropism mechanisms in plants^6^. Thus far, several organic compounds have been reported as gravitropic inhibitors during auxin response studies, including auxin transport and signaling. Examples of gravitropism inhibitors are 1-*N*-naphthylphthalamic acid (NPA)^7–9^, gravacin^10,11^, alkoxy-auxins^12^, auxinole^13,14^, and inhibitors of auxin biosynthesis, such as yucasin DF (Fig. 1a)^15^. The above-mentioned inhibitors are important chemicals for regulating plant physiology and potential herbicides. However, they are liable to suppress plant growth due to the inhibitory action of auxins. Therefore, compounds that selectively inhibit gravitropism without inhibiting plant growth need to be developed. These inhibitors can play a significant role in analyzing the gravitropism mechanism. In addition, these chemicals can contribute to the agrochemical industry through the development of plant growth regulators, such as weed suppressants, to keep plants green and as plant dwarfing agents.

Among auxin-related compounds, many studies have been conducted on auxin transport inhibitors as substances that affect gravitropism^6^. It has long been shown that natural plant compounds flavonoids such as naringenin and its precursors cinnamic acid, campferol, and quercetin, function as auxin transport inhibitors^16–18^. In particular, NPA is based on substances screened from the flavonoids that affect auxin transport^19^. Recently, in addition to flavonoids, NSAIDs, derivatives of the plant hormone salicylic acid, have been reported to inhibit auxin transport^20^.

*cis*-Cinnamic acid [*cis*-CA, Fig. 1b] is an active component of an allelochemical isolated from *Spiraea thunbergii*. *cis*-CA has also been reported to have inhibitory activity of auxin efflux^18^. We previously reported that *cis*-CA is localized at the root tip of lettuce by molecular imaging experiments using fluorescent molecular probes of *cis*-CA^21–24^. Based on these results, we subsequently focused on the effect of *cis*-CA on the gravitropism of plants and observed that it inhibits not only lettuce root elongation but also gravitropic bending of the roots, that is, *cis*-CA is a non-selective inhibitor of both elongation and gravitropic bending. Further studies revealed that among the synthetic analogs of *cis*-CA, ku-76 was a selective gravitropic bending inhibitor of lettuce roots at 5 μM; however, it did not suppress the root growth (Fig. 1c)^25^. Furthermore, we revealed that the (2*Z*,4*E*)-configuration of ku-76 comprising an aromatic ring and a carboxylic acid group is an essential structure of inhibitory activity^26^. We have designated ku-76 as a new lead compound for potent selective gravitropic bending inhibition.

In a previous report^27^, we synthesized stereochemically fixed bridged analogs of ku-76. Among the analogs, several 2′,4-bridged analogs, such as **1a** and **1b**, were found to be more potent gravitropic bending inhibitors (Fig. 1d), which were effective at less than 0.2 μM. Fixing the conformation can improve activity. Alternatively, the bridging structural transformation, including the C4-substituent effects such as conformational, steric, hydrophobic, and other electronic effects, may play a significant role in improving the inhibition activity. To examine this possibility, we synthesized and evaluated various C4-substituted synthetic analogs **2** to explore more potent inhibitors (Fig. 1e). Furthermore, we conducted studies on auxin regulation and gravitropic bending inhibition in *Arabidopsis thaliana* using a potent inhibitor; the inhibitor strongly inhibited gravitropism without inhibiting root elongation, indicating that it disrupts auxin distribution.

## Results

### Design of C4-halo analogs and their inhibitory activity for root gravitropic bending

**Figure 2:**
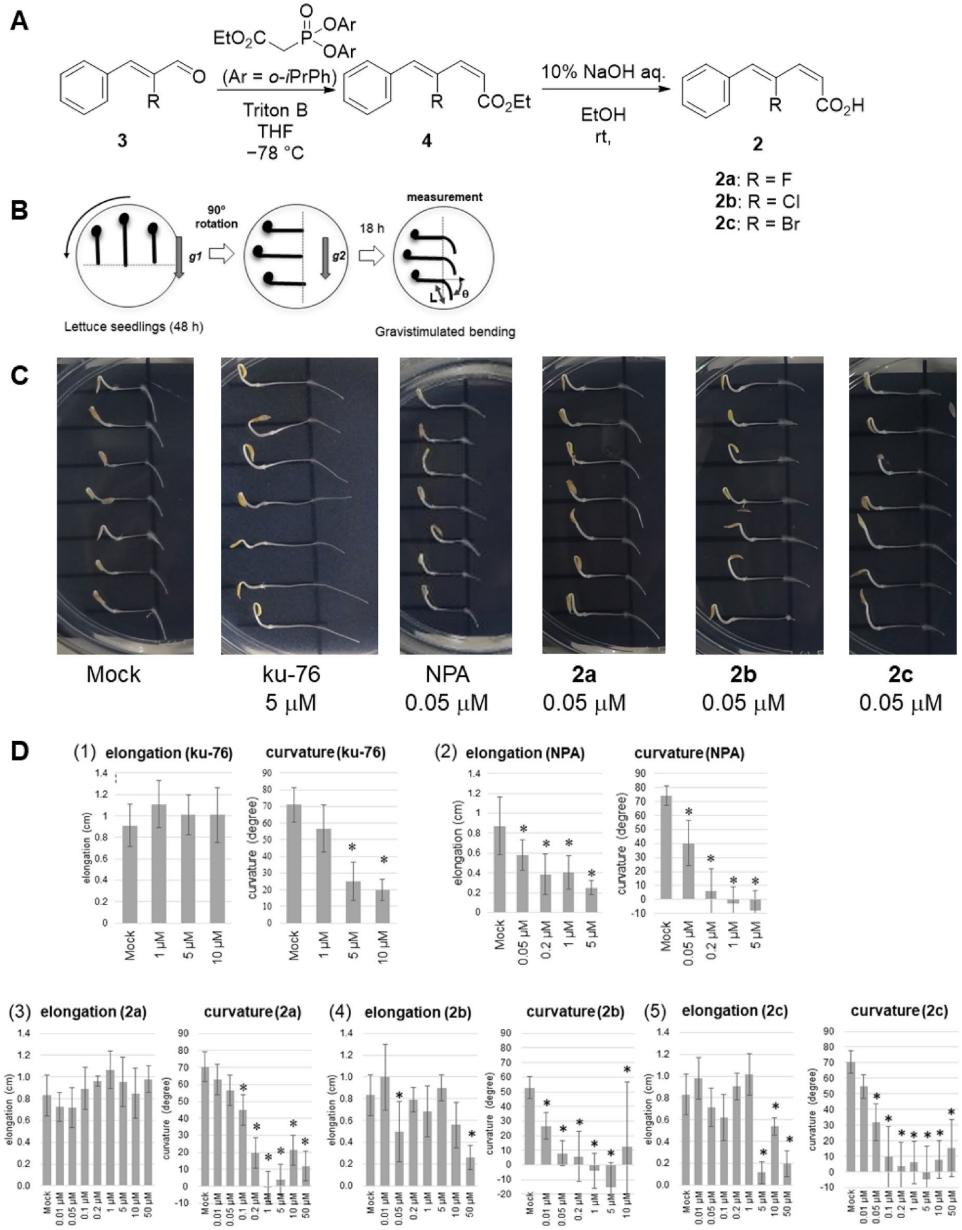
Gravitropic bending inhibitory tests of C4-halo analogs of ku-76 using lettuce roots. (**A**) Structure and synthetic procedure of C4-fluoro (**2a**), chloro (**2b**), and bromo (**2c**) ku-76 analogs. (**B**) Evaluation method of gravitropic bending inhibitory tests. (**C**) Representative images of an inhibitory test of gravitropic bending of Mock, 5 μM ku-76, 0.05 μM NPA, 0.05 μM **2a**, 0.05 μM **2b**, and 0.05 μM **2c**. (**D**) Inhibitory activity tests of gravitropic bending and elongation for dose–response relationship study of (1) ku-76, (2) NPA, (3) **2a**, (4) **2b**, and (5) **2c.** On the y-axis, the values of gravitropic bending (right) and elongation (left) represent mean ± standard deviation (SD). The asterisk indicates statistically significant differences between treatments and Mock at *p* < 0.05 (Turkey-Kramer’s test).

Because ku-76 inhibits root gravitropism but not root elongation, we attempted to synthesize ku-76 analogs that specifically inhibit root gravitropism at lower concentrations. We examined the effect of the C4-substituent when the conformation was not fixed, unlike in **1a** and **1b,** as shown in Fig. 1d. We synthesized ku-76 analogs with fluoro (**2a**), chloro (**2b**), and bromo (**2c**) groups as C4-substituents. Halogens are more hydrophobic; however, they do not cause steric hindrance. Thus, halogens would have little effect on conformation, but cell permeability would be better (Fig. 2A). The general synthetic method for the analogs **2a**–**2c** and other analogs described below was the *cis*-selective olefination of the corresponding aldehydes **3**, which was followed by hydrolysis of the resulting esters **4**, according to our previous report (Fig. 2A)^21^. The inhibitory tests for lettuce root growth and gravitropic bending were carried out according to the reported method (Fig. 2B and C)^26^. Ku-76 inhibited root gravitropism at concentrations above 1 μM, and no root growth inhibition was observed at 1 μM (Fig. 2D1). NPA, a known auxin transport inhibitor, inhibits root gravitropism by inhibiting the auxin transport. NPA inhibited root gravitropism at concentrations higher than 0.05 μM and also significantly inhibited root growth (Fig. 2D2). We observed that **2a**, **2b**, and **2c** inhibited root bending at 0.01 μM, and the highest inhibitory effect was observed at concentrations higher than 0.2 μM (Figs. 2D3–5). **2a** did not inhibit root growth in the evaluated concentration range, whereas **2b** and **2c** inhibited root growth at concentrations higher than 10 and 5 μM, respectively. These results indicate that although **2b** and **2c** inhibit root growth at high concentrations, **2a**, **2b**, and **2c** inhibit root gravitropism at lower concentrations than ku-76 and NPA. Furthermore, among these C4-halogenated analogs, **2a** was a more potent analog of ku-76 that does not inhibit root growth. Most gravitropic inhibitors exhibit inhibitory activity based on auxin transport, biosynthesis, and signal transduction, and they have been found to inhibit root growth. The C4 analogs of ku-76 inhibited gravitropism at low concentrations; however, they did not affect root growth, making them novel gravitropism inhibitors.

### Gravitropic bending inhibitory activity of 4-alkyl, aryl, and alkynyl C4-substituted analogs

**Figure 3:**
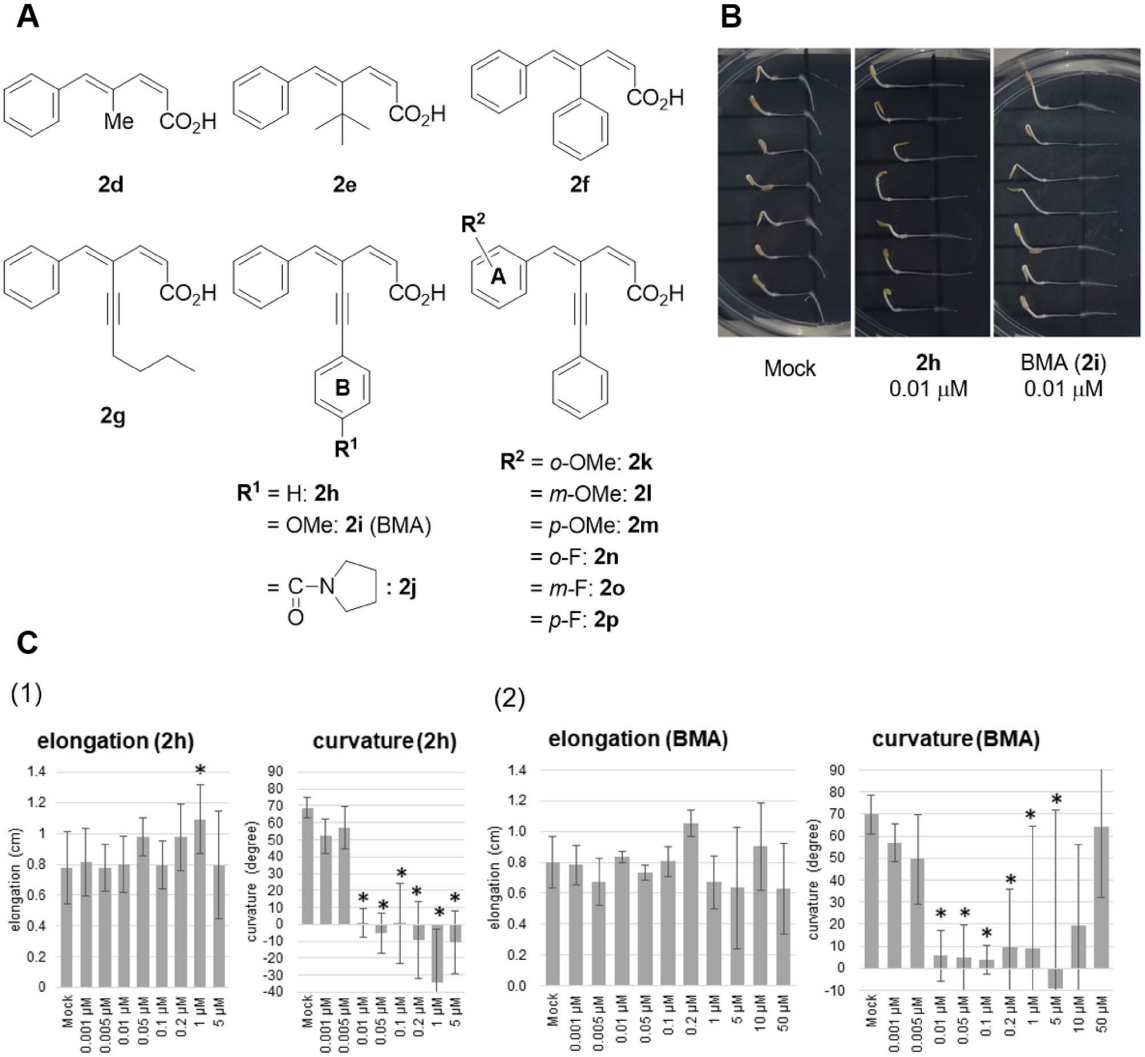
Gravitropic bending inhibitory tests of C4-carbon substituted analogs of ku-76 using lettuce roots. (**A**) Structure of synthetic compounds: methyl (**2d**), *tert*-butyl (**2e**), phenyl (**2f**), 1-hexynyl (**2g**), and phenylethynyl (**2h**–**2p**) compounds. (**B**) Representative images of inhibitory activity test of gravitropic bending for Mock, **2h**, and **2i** (BMA). (**C**) Inhibitory activity tests of gravitropic bending and elongation for dose–response relationship study for (1) **2h** and (2) **2i** (BMA). On the y-axis, the values of gravitropic bending (right) and elongation (left) represent mean ± SD. The asterisk indicates statistically significant differences between treatments and Mock at p < 0.05 (Turkey-Kramer’s test).

Because the C4-substituted analogs of ku-76 effectively inhibited root gravitropism, we synthesized C4-alkyl, aryl, and alkynyl analogs to explore more potent analogs (Figs. 3, S1). 4-Methyl analog **2d** was slightly more potent (< 1 μM) than ku-76, but elongation was relatively inhibited. The bioactivity of sterically hindered *tert*-butyl analog **2e** was less than that of **2d** and almost equivalent to that of ku-76. The bioactivity of 4-phenyl analog **2f** was similar to that of **2d**. Compared to these sp^2^- and sp^3^-carbon substituted analogs, alkynyl analogs exhibited stronger inhibitory activity. The 1-hexynyl analog **2g** inhibited gravitropic bending at less than 0.05 μM, albeit with scattered results. Furthermore, phenylethynyl analog **2h** was found to inhibit gravitropism at concentrations higher than 0.001 μM and completely inhibited gravitropism at 0.01 μM. Although phenylethynyl analog did not exhibit elongation inhibitory activity, the bending angle was less than 0° at 0.01 μM. Compared to the known inhibitor, NPA, which exhibited only weak bending inhibition at 0.05 μM apart from suppressing the elongation, **2h** is much more potent as a selective gravitropic bending inhibitor. Because the alkynyl group is sterically not bulky, the conjugated dienyl moiety would retain the *s-trans* conformation while enhancing lipophilicity, which may be important for inhibition. This remarkable result indicates that **2h** is the next leading compound for selective inhibition.

### Substituent effect on 2h analogs

As described above, **2h** is a novel and selective gravitropic bending inhibitor. To ensure the possibility of functionalization of this structure without loss of bioactivity, we examined the substituent effects on the A and B rings (Fig. 3A). *Para*-substitution with methoxy [**2i,** (*Z*)-4-((*Z*)-benzylidene)-6-(4-methoxyphenyl)hex-2-en-5-ynoic acid, BMA] or pyrrolidinocarbonyl (**2j**) groups on the B ring maintained the inhibitory activity even at 0.01 μM. In contrast, *ortho*- (**2k**), *meta*- (**2l**), and *para*-methoxy (**2m**) substitutions on the A ring significantly diminished the inhibitory activity. These results indicate that *para*-substitution on the B ring is an effective way to introduce functional groups into **2h** without the loss of bioactivity. However, it is difficult to modify the A ring of **2h** while maintaining its bioactivity. With the potent gravitropism inhibitors in hand, we then studied on the mechanism of inhibition using **2i** (BMA) as the strongest inhibitor.

### Evaluation of the effect of BMA on auxin signaling

When plants are subjected to gravistimulation, auxin is transported downward in the columella cells of the roots. The asymmetric auxin distribution in the tissue induces differential growth, causing the root to bend downward^1,28^. We focused on the function of auxin in positive gravitropism and investigated the effect of BMA on auxin regulation in *Arabidopsis thaliana*.

**Figure 4:**
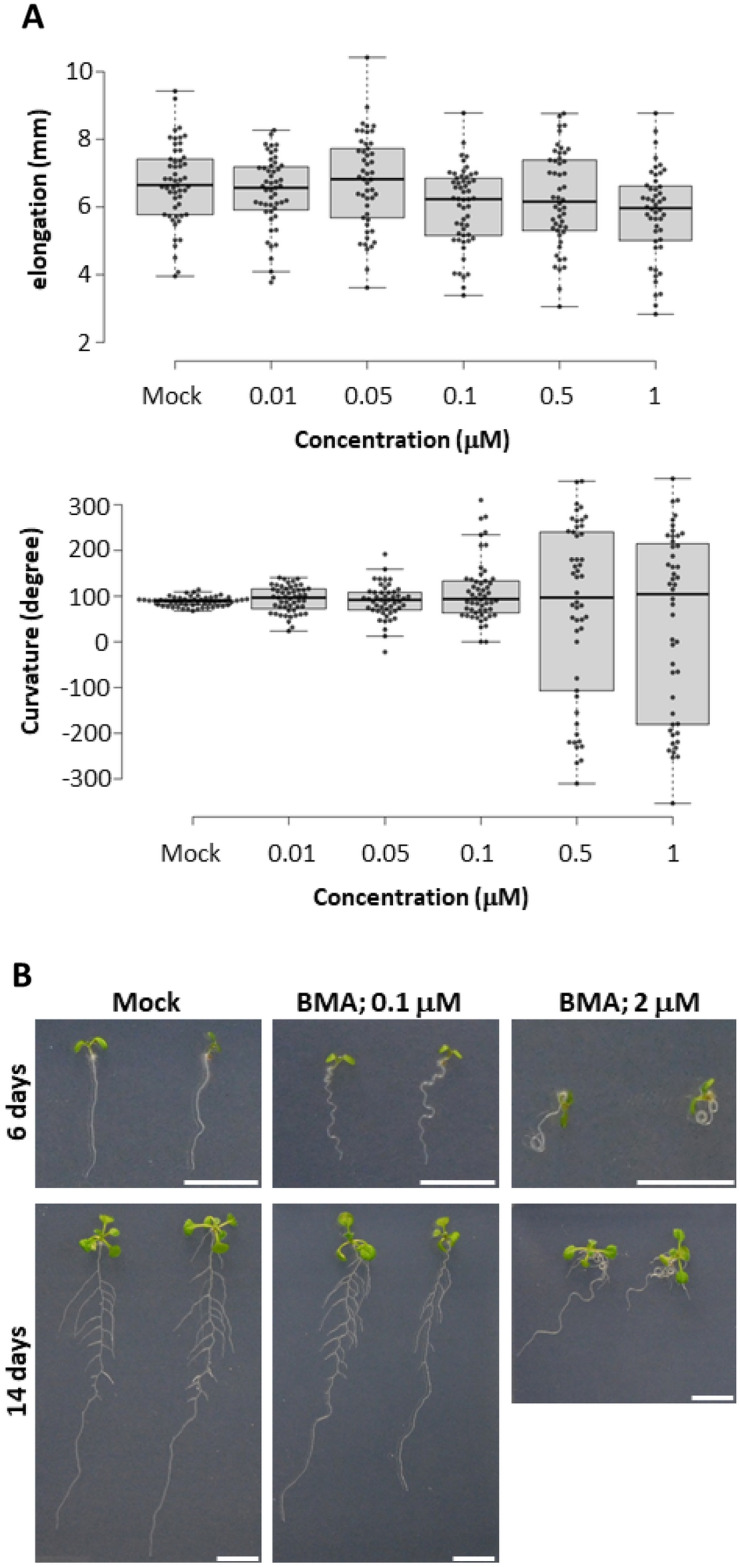
(**A**) Inhibitory activity tests of gravitropic bending and elongation for dose–response relationship study of BMA using Arabidopsis seedlings. Center lines show the medians; box limits indicate the 25th and 75th percentiles as determined by R software; whiskers extend 1.5 times the interquartile range from the 25th and 75th percentiles, outliers are represented by dots; width of the boxes is proportional to the square root of the sample size; data points are plotted as open circles. n = 53, 51, 49, 54, 50, 48 sample points. No significant differences in root elongation were found among all data (Tukey–Kramer, *P* < 0.01). (**B**) Effect of BMA on the growth of Arabidopsis seedlings. Arabidopsis seedlings were grown on a vertical agar plate containing 0.1 and 2 μM BMA for 6 and 14 days under continuous light conditions. Bar = 1 cm.

**Figure 5:**
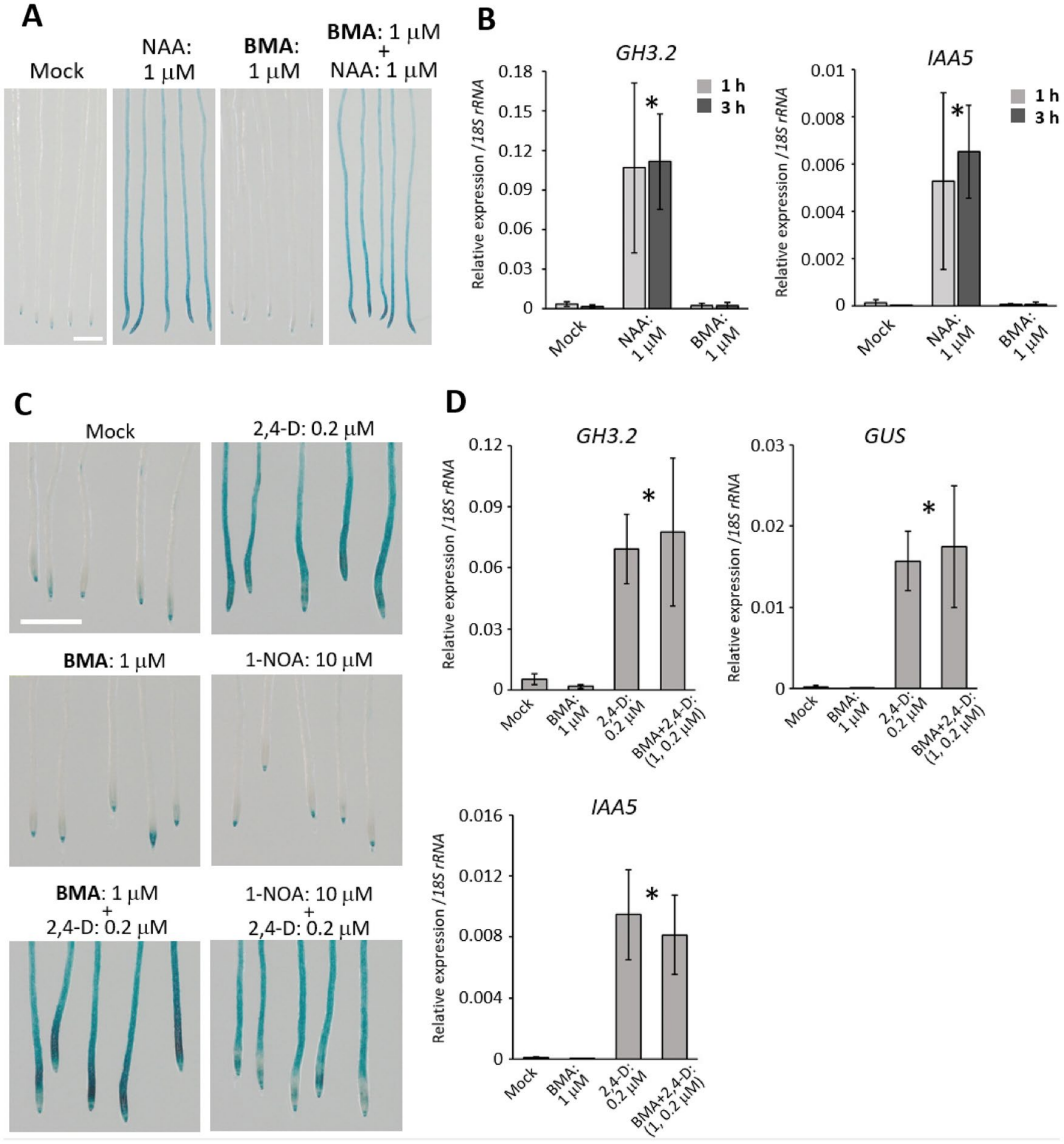
(**A**) Effect of BMA on *DR5:GUS* expression in Arabidopsis roots. Six-day-old seedlings of Arabidopsis with *DR5:GUS* plants were incubated for 7 h with 1 μM of BMA with or without 1 μM NAA. Bar = 1 mm. (**B**) The effect of BMA on the expression of the early auxin-inducible genes *GH3.2 and IAA5* in roots by qRT-PCR. The expression of indicated genes was quantified after 1 and 3 h after treatment. Error bar indicates S.D. Asterisks indicate no significant differences between samples (Tukey, *P* < 0.01). n = 4. (**C**) Evaluation of the effect of 1-NOA and BMA on 2,4-D uptake in Arabidopsis roots. Arabidopsis seedlings with *DR5:GUS* reporter system were preincubated for 1 h with 10 μM 1-NOA or 1 μM BMA. Subsequently, the seedlings were incubated for 7 h with 10 μM 1-NOA or 1 μM BMA, with or without 0.2 μM 2,4-D. Bar = 1 mm. (**D**) The effect of BMA on the expression of *GH3.2*, *IAA5* and *GUS* in roots by qRT-PCR. Arabidopsis seedlings with *DR5:GUS* reporter system were preincubated for 1 h with 1 μM BMA. Subsequently, the seedlings were incubated for 7 h with 1 μM BMA, with or without 0.2 μM 2,4-D. After treatment, the expression of indicated genes was investigated. Error bar indicates S.D. Asterisks indicate no significant differences between samples (Tukey, *P* < 0.01). n = 4.

First, we examined whether BMA affects gravitropism in Arabidopsis without inhibiting root elongation, as observed in experiments with lettuce (Fig. 4A). BMA did not inhibit root elongation at concentrations between 0.01 and 1 μM. Regarding the effect on gravitropism, BMA had a strong effect on root bending at concentrations higher than 0.1 μM. These results suggest that BMA affects gravitropism in Arabidopsis without inhibiting root elongation. When Arabidopsis seedlings were grown on vertical agar plates containing BMA, an abnormality in the direction of root growth was observed (Fig. 4B). When Arabidopsis was grown in the presence of 100 nM BMA, the roots showed a "wavy" phenotype compared to the mock control. In addition, in the presence of 2 μM BMA, the Arabidopsis roots exhibited a spiraling "curly" phenotype. In addition, lateral root formation was clearly observed in the 0.1 μM BMA treatment (Fig. 4B; 14 days). Furthermore, lateral root formation was also observed in the 2 μM BMA treatment, although it was difficult to observe due to root curling. In order to clarify the action of BMA, we first examined whether BMA exhibited auxin or anti-auxin activity using the auxin reporter system *DR5:GUS* (Fig. 5A). The synthetic auxin naphthaleneacetic acid (NAA) induced *DR5:GUS* expression, whereas BMA did not. In addition to the *DR5:GUS* assay, we examined whether BMA induces the expression of the auxin-inducible genes *GH3.2* and *IAA5* by qRT-PCR analysis. It has already been reported that *GH3.2* and *IAA5* respond to high levels of auxin by qRT-PCR analysis. Expression of *GH3.2* and *IAA5* was induced at 1 h and 3 h after NAA treatments, whereas BMA did not induce expression of these genes (Fig. 5B). Anti-auxins, such as auxin receptor TIR1/AFBs inhibitors, suppress the induction of expression of auxin-responsive genes including *DR5:GUS* reporter^13^. When BMA was co-treated with NAA, BMA did not inhibit *DR5:GUS* expression induced by NAA (Fig. 5A). These results indicate that BMA does not exhibit auxin or anti-auxin activity.

### Evaluation of the effect of BMA on auxin transport

The differential distribution of auxin occurs before differential tissue and organ growth during gravitropism^29^. The differential distribution of auxin is due to the action of auxin influx carrier proteins, such as AUX1, and auxin efflux carrier proteins such as the PIN^29,30^. Therefore, we first examined the effect of BMA on auxin influx. Previous studies have shown that 1-naphthoxyacetic acid (1-NOA) inhibits auxin uptake by inhibiting the AUX1 auxin influx carriers. The synthetic auxin 2,4-dichlorophenoxyacetic acid (2,4-D) induces the expression of the auxin reporter *DR5:GUS*, and 1-NOA cancels the *DR5:GUS* expression induced by 2,4-D^31^. We examined the effect of 1-NOA on the *DR5:GUS* expression induced by 2,4-D and confirmed that 10 μM of 1-NOA suppressed the *DR5:GUS* expression induced by 2,4-D (Fig. 5C). When Arabidopsis seedlings were co-treated with 1 μM BMA, a concentration that sufficiently inhibits root gravitropism in Arabidopsis, and 2,4-D, BMA did not suppress the *DR5:GUS* expression induced by 2,4-D, indicating that BMA possibly did not affect auxin influx (Fig. 5C). When 2,4-D were co-treated with BMA, expression of *GH3.2*, *IAA5* and *GUS* in the *DR5:GUS* reporter system was induced as in 2,4-D treatment, which also supports that BMA did not inhibit 2,4-D uptake (Fig. 5D).

**Figure 6:**
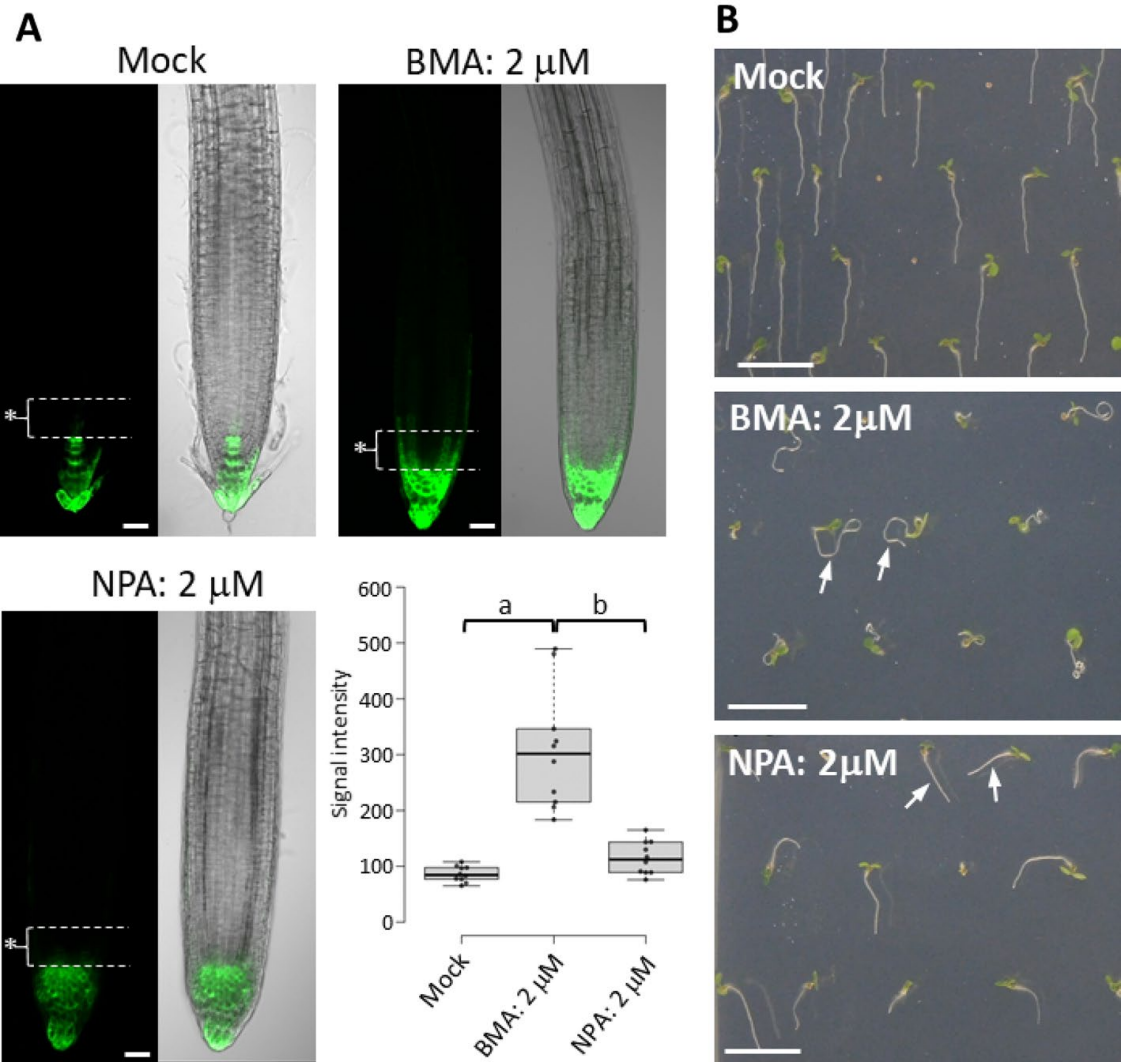
(**A**) Effect of BMA, and NPA, on *DR5:GFP* expression in Arabidopsis root tips. Arabidopsis *DR5:GFP* transgenic line was grown on a vertical agar plate containing 2 μM of BMA and NPA for 6 days under contentious light conditions. GFP signals of the seedlings were observed using LSCM Olympus FV-1000. Bar = 30 μm. Fluorescence signal intensity in the ROI (Asterisks; region 50 μm from QC) were compared. (a) and (b) indicate significant difference between data (Tukey, *p* < 0.01). n = 10. (**B**) Comparison of the effects of BMA and NPA on the growth of Arabidopsis seedlings. Arabidopsis was grown on a vertical agar plate containing 2 μM of BMA and NPA for six days under contentious light conditions. Arabidopsis roots are elongated in a curve in the presence of BMA (arrows in the BMA image), whereas roots are elongated linearly in the presence of NPA (arrows in the NPA image). Bar = 1 cm.

Recently, crystal structure analysis of the PIN protein showed that NPA inhibits auxin transport by binding to PIN auxin efflux carrier proteins^8,9^. NPA induces auxin accumulation in root tips by inhibiting auxin transport, resulting in the accumulation of *DR5:GFP* signal in the root tips of NPA-treated roots (Fig. 6A). We investigated whether BMA showed a similar effect on *DR5:GFP* expression in the root tips as NPA (Fig. 6A). Treatment with BMA induced strong GFP signals in the lateral root cap, epidermis, and cortex, basal to the root tip (asterisks in Fig. 6A). The pattern of *DR5:GFP* expression was different from that observed in NPA-treated roots. These results indicate that BMA affects auxin transport at the root tip and inhibits gravitropism by affecting auxin distribution at the root tip in a manner different from that of NPA.

When Arabidopsis seedlings were grown on a vertical agar plate containing BMA, the seedlings showed "wavy" and "curly " root phenotypes, which were not observed when grown in the presence of NPA. Arabidopsis roots elongated linearly in the presence of NPA (Fig. 6). In root hair formation, NPA promoted the formation of excessive root hairs, whereas BMA did not cause excessive root hair formation as NPA did (Fig. S3A and B). The effects of NPA and BMA were further compared using etiolated seedlings (Fig. S3C–E). Both NPA and BMA significantly inhibited hypocotyl elongation (Fig. S3D). On the other hand, NPA significantly affected the direction of hypocotyl elongation, while BMA did not (Fig. S3E). These results indicate that BMA is a novel inhibitor with a different mechanism of action than that of NPA. The PIN proteins that play important roles in root gravitropism are PIN3, which determines the direction of auxin transport in columella cells, and PIN2, which transports auxin from the root tip to the basal side. The root phenotype caused by BMA treatment implies that they are gravity-insensitive, similar to the *pin2* mutant^32^. Therefore, we evaluated the effect of BMA on the function of PIN2. Because PIN proteins determine the direction of auxin transport by polar intracellular localization, we observed the effect of BMA on the subcellular localization of PIN2-GFP (Fig. S4). The results showed that the localization pattern of BMA-treated PIN2-GFP was similar to that of untreated PIN2-GFP, indicating that BMA does not affect the localization of PIN2-GFP. Because BMA inhibits gravitropism but does not affect growth, it may work specifically in columella cells, which are gravity-sensitive cells. Therefore, we examined whether BMA affected the localization of PIN3, which confers gravitropism in columella cells^30^. We found that BMA treatment did not affect the expression of PIN3-GFP (Fig. S4D). Thus, it is unlikely that BMA is involved in auxin transport by affecting the PIN2 localization pattern and PIN3 expression. In the future, more detailed mechanisms of action of BMA will be elucidated when the target proteins of BMA have been identified.

## Conclusion and perspectives

Considering ku-76 as a lead compound for selective gravitropism inhibition, C4-substituted analogs were designed, synthesized, and evaluated for gravitropic bending and elongation inhibitory activity using lettuce roots. The C4-halo-analogs **2a–c** were 50 times more potent than ku-76. These analogs inhibited the gravitropic bending at 0.1 μM without substantially affecting elongation. The analogs with 4-methyl- (**2d**) and phenyl- (**2f**) substituents were slightly more potent than ku-76, whereas those with the sterically hindered *tert*-butyl substituent (**2e**) were relatively less potent. Although the analog with 4-hexynyl substituent exhibited moderate activity, 4-phenylethynyl analog **2h** exhibited the most potent activity even at only 10 nM among the synthetic analogs, including **1a** and **1b**, as described in a previous report. This gravitropic bending inhibitory activity, without inhibition of elongation, was much stronger than that of the known inhibitor NPA. Although substitution on the A-ring of **2h** critically diminished the activity, the *para*-substitution on B-ring was tolerated, indicating the possibility of functionalization of **2h** at the *para*-position without significant loss of activity. These results demonstrated that **2h** and 4-(*p*-methoxyphenylethynyl) analog (BMA) are second-generation lead compounds for the selective inhibition of gravitropic bending. These highly potent analogs are not only useful tools for elucidating the mechanism of gravitropism. In addition, they provide significant information for the development of novel, non-toxic, and environment-friendly agrochemicals, such as weed suppressants, while keeping plants green and functioning as plant dwarfing reagents.

Auxin plays an important role in gravitropism in plants. Several auxin-related inhibitors have been reported to inhibit gravitropism, and among them, auxin transport inhibitors effectively inhibit gravitropism. In this study, evaluation using Arabidopsis indicated that BMA also affected gravitropism, probably by affecting auxin transport. The effects of BMA on Arabidopsis phenotypes and *DR5:GFP* expression patterns indicated that BMA may be a novel inhibitor that differs in action from previously reported auxin transport inhibitors.

Recently, following the determination of the structure of PIN proteins, NPA was shown to inhibit auxin transport by binding directly to PIN proteins^8,9^. This indicates that compounds that universally inhibit the auxin efflux activity of PIN interfere not only with gravitropism but also with tissue growth. Because the BMA synthesized in this study specifically affected gravitropism, it is unlikely that it directly inhibits PIN throughout the plant; however, it affected auxin transport only at the root tips. In particular, BMA appears to strongly affect lateral auxin transport in response to recognizing the direction of gravity.

To estimate the target of BMA, we compare BMA with previously reported auxin transport inhibitors. It has been reported that *cis*-CA, the origin of BMA, inhibits auxin efflux^18^. However, *cis*-CA differs from the action of BMA in many ways: it is effective at high concentrations, significantly inhibits root growth, induces *DR5:LUC* expression, and promotes lateral root formation. Therefore, together with the fact that *cis*-CA and BMA have very different structures, it is likely that *cis*-CA and BMA have different mechanisms of action. However, because *cis*-CA exhibits a wide variety of actions, it may share some similarities with BMA if the focus is on auxin transport. Regarding the possibility that BMA targets the ABCB auxin transporters, we compare BMA to gravacin, which has been reported as an inhibitor of ABCB19^11^. Gravacin has been shown to inhibit gravitropism of etiolated hypocotyl by inhibiting ABCB19. BMA differs from the action of gravacin in that BMA has little effect on the gravitropic response of the hypocotyl. BFA and Endosidin have been shown to inhibit auxin transport by acting on intracellular vesicle transport and affecting the localization of auxin transport proteins^6^. Upon treatment with these compounds, PIN2-GFP has been shown to form intracellular aggregates. On the other hand, no intracellular aggregates of PIN2-GFP or PIN3-GFP were formed when treated with BMA. Thus, it appears that BMA does not affect auxin transport by intracellular aggregation of PIN2 and PIN3 at least. The Arabidopsis phenotype after BMA treatment is similar to the phenotype exhibited by the *pin2* or *aux1* mutants. Although this study suggests that BMA is not involved in the localization of PIN2-GFP and may not be involved in auxin influx, more detailed analysis using *pin2* and *aux1* mutants and over-expressors, as well as biochemical analysis of auxin transport activity, is expected.

Recently, it was reported that the LZY-RLD-mediated signal transduction plays an important role in gravity signaling that bridges the link between amyloplast sedimentation and changes in the direction of auxin transport^33^. In lateral root columella cells, LZY3 polarly localizes in the plasma membrane on the gravity side of the cell and recruits RLD1 in the plasma membrane through physical interaction. RLD1 is expected to determine the polarity of auxin transporters through the regulation of membrane trafficking. The possibility that BMA may affect gravitropism specifically by acting somewhere in the signaling processes, such as the polar localization of LZY, the interaction between LZY and RLD, and the activity of RLD1, cannot be excluded and is a subject of interest.

It has been shown that not only the mechanism determining the subcellular localization of PIN but also the regulation of auxin efflux activity by phosphorylation-dephosphorylation is important in determining the direction of auxin transport^34^. Among kinases in plants, AGC-protein kinase families such as PID and D6PK regulate the activity of PIN by phosphorylation and are involved in gravity- and photo-tropisms^35,36^. Although the mechanism of regulation of the polarity of auxin transport activity by kinases is unclear, it would be interesting to analyze if BMA is involved in regulating such auxin transport activity specifically in gravity-sensing cells.

## Experiments

### Methods

^1^H and ^13^C NMR spectra were recorded on a JEOL JNM EX-270 (270 and 67.5 MHz), JNM AL-400 (400 and 100 MHz), and a JNM ECA-600 (600 and 150 MHz) spectrometers. The IR spectra were recorded on a SHIMADZU IRPrestige-21 FT-IR spectrophotometer using a KBr disk or NaCl cell. Mass spectra were obtained using a JEOL JMS-700 or JMS-T100CS. High-resolution mass spectra were obtained using a JEOL JMS-700 or JMS-T100CS.

### Representative procedure for the synthesis of (2*Z*,4*Z*)-4-substituted-5-phenylpenta-2,4-dienoic acid

#### Synthesis of (2*Z*,4*Z*)-4-fluoro-5-phenylpenta-2,4-dienoic acid (2a)

To a solution of (*Z*)-2-fluorocinnamaldehyde (695 mg, 4.57 mmol) in THF (10 mL), Triton B (40% MeOH solution, 2.7 mL, 7.0 mmol) was added at − 78 °C under argon atmosphere. After stirring for 15 min, ethyl 2-[bis(2-isopropylphenoxy)phosphoryl] acetate (2.22 g, 5.49 mmol) in THF (8.0 mL) was added to the mixture. After stirring the resulting mixture for 2 h at − 78 °C, the reaction was quenched with saturated aqueous NH_4_Cl. The mixture was then extracted with EtOAc. The combined organic layers were washed with brine, dried over MgSO_4_, filtered, and concentrated *in vacuo*. The crude product was purified by silica gel column chromatography (hexane/EtOAc = 9/1) to obtain ethyl ester of **2a** (650 mg, 65%, *Z*:*E* =  > 99:1) as a pale-yellow oil.

To a solution of the ester of **2a** (542 mg, 2.46 mmol) in EtOH (8.0 mL), 10% NaOH_aq_ (8.0 mL) was added at room temperature. After stirring for 1 h, the mixture was washed with hexane. After acidifying the water layer with 3M HCl, the mixture was extracted using EtOAc. The combined organic layers were washed with brine, dried over MgSO_4_, filtered, and concentrated *in vacuo*. The crude product was recrystallized from hexane to obtain **2a** (433 mg, 92%).

### Plant materials and growth condition

In this study, we used *Lactuca sativa* cv. Great Lakes 366 (Lettuce), which was unrestricted commercially bought non-transgenic seeds (Atariya Noen Co. LTD, Chiba, Japan), and *Arabidopsis thaliana* accession Columbia-0 (Col-0) (ABRC (Arabidopsis Biological Resource Center, The Ohio State University) as wild-type lines. No permit was needed for use and the research followed relevant institutional and national guidelines and legislation. The transgenic lines of *Arabidopsis thaliana* (DR5p:GUS, DR5p:GFP, PIN1-GFP, PIN2-GFP, and PIN3-GFP) used in this study were in the Col-0 background.

Lettuce seeds (*Lactuca sativa* cv. Great Lakes 366) were incubated on a solidified agar (2% w/v) in a Petri dish where they were allowed to germinate and grow at 25 °C for 48 h in the dark.

Surface-sterilized Arabidopsis seeds were sown on MS plates (0.5 × Murashige Skoog salts, 1% [w/v] sucrose, 0.05% [w/v] MES, and 2% [w/v] agar, pH 5.7), incubated in the dark at 4 °C for 2–3 days, and grown at 23 °C in a growth chamber under continuous light. For experiments using etiolated seedlings, the seedlings were grown in the dark for 3 days.

### Inhibitory test for gravitropic bending using lettuce seedlings

Well-grown lettuce seedlings (5–7 pieces) were transferred to solidified agar plates (1% [w/v]) containing test compounds (50 μM) in a single plate, and they were arranged parallel to the gravity vector. The seedlings were pre-incubated vertically under the same conditions for 1 h, and then gravistimulated by reorienting the plates to 90° and incubated for 18 h. Subsequently, root images were captured using a digital camera, and the angles of gravitropic curvature and the length of the roots were analyzed using ImageJ software (ver. 1.53 m, https://imagej.nih.gov/ij/download.html). For the statistical evaluation, the Tukey–Kramer test was performed using R software (ver. 4.2.2, https://cran.r-project.org/) (Figs. 2, 3, S1 and S2).

### Elongation and gravitropism analysis using Arabidopsis

Vertically grown four-day-old seedlings were transferred to the MS plates containing chemicals and were incubated for two days at 23 °C under continuous light. The elongation length of the roots for two days after the transfer was measured using ImageJ. For gravitropism, vertically grown seven-day-old seedlings were transferred to MS plates containing chemicals and incubated for 1 h in the dark. The MS plate was then reoriented to 90° and incubated for 24 h in the dark. The root tip angles were measured using ImageJ. Length and growth direction of etiolated hypocotyl were observed by measuring the length of the hypocotyl and the direction of the hypocotyl tip using ImageJ (Figs. 4 and 5).

### *DR5p:GUS* reporter assay

Five-day-old *DR5::GUS* seedlings were preincubated in germination medium [1/2 × MS, 1% (w/v) sucrose, 0.01% (w/v) myo-inositol, 0.05% MES-KOH (pH 5.7)] containing chemicals for 1 h. Subsequently, the seedlings were incubated in germination medium containing chemicals with or without 1 μM NAA or 0.2 μM 2,4-D for 7 h. After incubation, the seedlings were incubated in GUS staining buffer (100 mM sodium phosphate (pH 7.0), 10 mM EDTA, 10 mM ferricyanide, 10 mM ferrocyanide, 0.1% Triton X-100) containing 1 mM 5-bromo-4-chloro-3-indolyl β-d-glucuronide (Glicosine) at 37 °C until sufficient staining was observed. Samples were observed under a digital microscope (DSX110; Olympus).

### qRT-PCR analysis

Roots of 25-30 Arabidopsis plants treated with each compound for the indicated times were collected and total RNA was isolated using RNeasy Plant Mini Kit (QIAGEN). The cDNA was prepared using PrimeScriptII Reverse Transcriptase (TaKaRa) and RNase-Free DNase Set (QIAGEN) according to the manufacture’s instruction. The SYBR Premix Ex TaqII (TaKaRa) was used for the preparation of real-time qPCR mix, and then real-time qPCR was performed using the LightCycler 96 real-time PCR system (Roche). Primers used in this study were listed (Table S8).

### *DR5p:GFP* reporter assay

Arabidopsis seedlings expressing *DR5p:GFP* were grown vertically on MS plates containing chemicals for 4 days. GFP fluorescence was observed, and images were obtained using a confocal laser scanning microscope (FV-1000; Olympus). For quantification of the GFP signal, the average intensity of the fluorescent signal was determined using the region 50 μm above the QC of the root as the ROI using Olympus Fluoview Ver4.2 software.

## Supplementary Information

Supplementary Information.

## Data Availability

All data generated or analyzed during this study are included in this published article and its supplementary information files.
