# Supplementary material for: Design and synthesis of strong root gravitropism inhibitors with no concomitant growth inhibition
Source: Sci Rep. 2023 Mar 30;13:5173. doi: 10.1038/s41598-023-32063-z (PMC10063617; doi:10.1038/s41598-023-32063-z)
Supplement: Supplementary file 1 — Supplementary Information. [file 41598_2023_32063_MOESM1_ESM.docx]

**Supporting Information**

**Design and synthesis of strong root gravitropism inhibitors with no concomitant growth inhibition**

Takeshi Nishimura,^d^ Saki Makigawa,^b^ Jun Sun,^b^ Kozue Kodama,^a^ Hiromi Sugiyama,^a^ Kenji Matsumoto,^a^ Takayuki Iwata,^a^ Naoya Wasano,^c^ Arihiro Kano,^a^ Miyo Terao Morita,^d^ Yoshiharu Fujii,^c^ Mitsuru Shindo,^a^*

Contents

1. General remarks S2

2. Synthetic procedures S3

2-1. Preparation of α-substituted cinnamaldehydes, intermediates for analogues **2a-g** S3

2-2. 2-2. 4-Arylethynyl analogues **2a-2g** S5

2-3. Synthesis of 4-arylethynyl analogues **2h-j** S7

2-4. Synthesis of analogues **2k-p** S12

3. 1H and 13C NMR spectra S22

4. Figure S1 S32

5. Figure S2 S33

6. Figure S3 S34

7. Figure S4 S35

8. Raw data of gravitropism bending inhibition tests S36

9. Primers used in this study. S46

10. References S47

**1. General remarks**

The ^1^H and ^13^C NMR spectra were recorded on a JNM EX-270 (270 and 67.5 MHz), AL-400 (400 and 100 MHz), and a JNM ECA-600 spectrometer (600 and 150 MHz). Chemical shifts were reported in ppm downfield from the peak of Me_4_Si (TMS) used as the internal standard. Splitting patterns are designed as “br, s, d, t, q, and m,” indicating “broad, singlet, doublet, triplet, quartet, and multiplet,” respectively. The IR spectra were recorded on a SHIMADZU IRPrestige-21 FT-IR spectrophotometer using a KBr disk or a NaCl cell. Mass spectra were obtained on a JEOL JMS-700 or a JEOL JMS-T100CS. High-resolution mass spectra were obtained on a JEOL JMS-700 or a JEOL JMS-T100CS. Column chromatography was performed on silica gel (Kanto Chemical Co.). Thin-layer chromatography was performed on pre-coated plates (0.25 mm, silica gel Merck 60 F254). Reaction mixtures were stirred magnetically. We use common software including ImageJ (ver. 1.53m) and Olympus microscope operating software. We do not use any software that requires special citation.

**2. Synthetic procedures**

**2-1. Preparation of α-substituted cinnamaldehydes, intermediates for analogues 2a-g**

R = Cl, Br, Me (for **2b-d**): Commercially available

**(*Z*)-2-Fluoro-3-phenylacrylaldehyde** (**3a**, R = F, for **2a**)

To a solution of (*Z)*-ethyl 2-fluoro-3-phenylacrylate (1.2 g, 6.1 mmol)^1^ in$\mathrm{THF}$ (25 mL) was cooled to at -78 °C under argon, added DIBAL (14.7 mL, 14.7 mmol). After being stirred for 2 h, the reaction was quenched with H_2_O, extracted with CH_2_Cl_2_ (40 mL × 3), and the organic layer was washed with 1M HCl aq, and brine, and dried over MgSO_4_. The crude product was purified by silica gel column chromatography (20% ethyl acetate in hexane) to give 695 mg (75%) of (*Z)-*2-fluoro-3-phenylprop-2-en-1-ol as colorless solid: ^1^H-NMR (270 MHz, CDCl_3_) δ: 4.30 (2H, dd, *J* = 6.8, 14.3 Hz), 5.79 (1H, d, *J* = 38.9 Hz), 7.23-7.53 (5H, m).

To a solution of (*Z*)*-*2-fluoro-3-phenylprop-2-en-1-ol (695 mg, 4.57 mmol) in CH_2_Cl_2_ (30 mL) was added MnO_2_ (11.9 g, 137 mmol). After being stirred for 4 h, the mixture was purified by using celite. The crude product was 912.5 mg of (*Z*)-2-fluoro-3-phenylacrylaldehyde (**3a**) as yellow oil, which was used without further purification. ^1^H-NMR (270 MHz, CDCl_3_) δ: 6.63 (1H, d, *J_HF_* = 34.3 Hz), 7.45-7.74 (5H, m), 9.37 (1H, t, *J_HF_* = 16.7 Hz); The spectral data were consistent with those reported in the literature.^2^

**Synthesis of (*E*)-2-Benzylidene-3,3-dimethylbutanal** (R = *t*-Bu, for **2e**)

**(*E*)-2-Benzylidene-3,3-dimethylbutanoic acid**^3^

 To a solution of ethyl 2,2-dibromo-3,3-dimethylbutanolate (2.1 g, 7.0 mmol) in THF (20 mL), cooled to -78 °C under argon, was added *t-*BuLi (17.8 mL in pentane, 28.5 mmol). After stirring for 30 min, the mixture was allowed to warm to 0 °C. After another 1 h, the resulting reaction mixture was allowed to warm to room temperature and then a solution of benzaldehyde (531 mg, 5.00 mmol) in THF was added. After stirring for 2.5 h, the reaction mixture was quenched with 1M NaOH aq and washed with hexane. The water layer was acidified with 3M HCl and the mixture was extracted with EtOAc. The combined organic layer was washed with brine, dried over MgSO_4_, filtered and concentrated *in vacuo*. The crude yellow oil (701.8 mg) was used in the next reaction without further purification: ^1^H-NMR (270 MHz, CDCl_3_) δ: 1.15 (9H, s), 7.19-7.38 (5H, m), 7.44 (1H, s).

**(*E*)-2-Benzylidene-3,3-dimethylbutan-1-ol**

To a suspension of LiAlH_4_ (261 mg, 6.88 mmol) in Et_2_O (20 mL) at 0 °C under argon was added a solution of (*E*)-2-benzylidene-3,3-dimethylbutanoic acid (702 mg, 3.44 mmol) in Et_2_O (15 mL). The mixture was stirred at room temperature for 4 h. After the resulting mixture was cooled to 0 °C, the reaction was quenched with H_2_O (1 mL), 1M NaOH (1 mL), H_2_O (1 mL). The mixture was warm to room temperature and stirred for 30 min. The precipitate was filtered through celite and the filtrate was concentrated *in vacuo*. The crude product was purified by silica gel column chromatography (Et_2_O in hexane, 10% to 20%) to give 228 mg (35%) of (*E*)-2-benzylidene-3,3-dimethylbutan-1-ol as light yellow oil: ^1^H-NMR (270 MHz, CDCl_3_) δ: 1.03 (9H, s), 4.29 (2H, s), 6.72 (1H, s), 7.13-7.30 (5H, m).

**(*E*)-2-Benzylidene-3,3-dimethylbutanal (3e)**

To a solution of (*E*)-2-benzylidene-3,3-dimethylbutan-1-ol (228 mg, 1.20 mmol) in CH_2_Cl_2_ (20 mL) was added MnO_2_ (3.1g, 36 mmol). After being stirred for 3 h, the mixture was filtered through celite. The crude product (light yellow oil) was used to the next reaction without further purification: ^1^H-NMR (400 MHz, CDCl_3_) δ: 1.14 (9H, s), 7.18-7.35 (5H, m), 7.39 (1H, s), 9.52 (1H, s).

**Synthesis of (*E*)-2,3-diphenylacrylaldehyde** (**3f**, R = Ph, for **2f**)

To a solution of α-bromocinnamaldehyde (1.06 g, 5.02 mmol) in toluene and H_2_O (7:3, 17 mL) was added Pd(PPh_3_)_4_ (116 mg, 0.100 mmol), PhB(OH)_2_ (671 mg, 5.50 mmol) and Na_2_CO_3_ (2.01 g) under argon atmosphere. The mixture was warmed to 100 °C and stirred for 5 h. The resulting mixture was filtrated through celite. The filtrate was extracted with EtOAc. The combined organic layer was washed with brine, dried over MgSO_4_, filtered and concentrated *in vacuo*. The crude product was purified by silica gel column chromatography (20% CH_2_Cl_2_ in hexane) to give 429 mg (41%) of (*E*)-2,3-diphenylacrylaldehyde as yellow oil. ^1^H-NMR (270 MHz, CDCl_3_) δ: 7.18-7.52 (11H, m), 9.78 (1H, s); The spectral data were consistent with those reported in the literature.^4^

**(*E*)-2-Benzylideneoct-3-ynal**^1^ (**3g**, R = 1-hexynyl, for **2g**)

To a solution of 1-hexyne (616 mg, 7.50 mmol) in benzene (25 mL) was added α-bromocinnamaldehyde (1.06 g, 5.02 mmol), Pd(PPh_3_)_4_ (231 mg, 0.200 mmol), CuI (95 mg, 0.50 mmol) and diisopropylamine (1.4 mL, 10 mmol) at room temperature under argon atmosphere. After the mixture was stirred for 6 h, the resulting solution was diluted by hexane and washed with brine, dried over MgSO_4_, and concentrated *in vacuo*. The crude product was purified by silica gel column chromatography (hexane/ether 95:5) to provide 713 mg (67%) of **3g** as brown oil; ^1^H-NMR (270 MHz, CDCl_3_) δ: 0.97 (3H, t, *J* = 7.3 Hz), 1.45-1.73 (4H, m), 2.57 (2H, t, *J* = 6.8 Hz), 7.42-8.12 (6H, m), 9.56 (1H, s).

**2-2. 4-Arylethynyl analogues 2a-2g**

**(2*Z*,4*Z*)-4-fluoro-5-phenylpenta-2,4-dienoic acid (2a)**

Colorless needles (hexane, mp. 85–87 °C); ^1^H-NMR (400 MHz, CDCl_3_) δ: 5.90 (1H, d, *J* = 13 Hz), 6.09 (1H, d, *J*_H-F_ = 36 Hz), 6.30 (1H, dd, *J* = 13 Hz, *J*_H-F_ = 28 Hz), 7.28–7.60 (5H, m); ^13^C-NMR (150 MHz, CDCl_3_) δ: 117.1 (CH, d, *J*_C-F_ = 8.7 Hz), 119.0 (CH), 128.6 (2CH), 129.6 (CH, *d, J*_C-F_ = 8.7 Hz), 131.7 (CH, *J*_C-F_ = 26.0 Hz), 132.6 (C, d*, J*_C-F_ = 2.9 Hz), 154.5 (C, d*, J*_C-F_ = 263 Hz), 171.6 (C); IR (KBr) 1697 cm^-1^; EIMS *m/z* 192 (M^+^), 147 (100%); Anal. calcd for C_11_H_9_FO_2_: C, 68.58; H, 4.67, found: C, 68.75; H, 4.72.

**(2*Z*,4*Z*)-4-Chloro-5-phenylpenta-2,4-dienoic acid (2b)**

Colorless needles (5% CH_2_Cl_2_ in hexane); mp. 108.4–110.0 °C; ^1^H-NMR (400 MHz, CDCl_3_) δ: 5.91 (d, *J* = 12 Hz, 1H), 6.58 (d, *J* = 12 Hz, 1H), 7.08 (s, 1H), 7.33–7.40 (m, 3H), 7.70 (d, *J* = 7.2 Hz, 2H); ^13^C-NMR (100 MHz, CDCl_3_) δ: 119.9 (CH), 125.3 (C), 128.3 (CH), 128.9 (CH), 129.7 (CH), 132.6 (CH), 134.0 (C), 140.8 (CH), 171.2(C); IR (KBr): 1693 cm^-1^. MS (EI) m/z: 208 (M^+^); Anal. Calcd for C_11_H_9_ClO_2_: C, 63.32; H, 4.35. Found: C, 63.22; H, 4.35.

**(2*Z*,4*Z*)-4-Bromo-5-phenyl-penta-2,4-dienoic acid (2c)**

Colorless needles (5% CH_2_Cl_2_ in hexane); mp. 64.3–65.5 °C; ^1^H-NMR (400 MHz, CDCl_3_) δ: 5.82 (d, *J* = 12 Hz, 1H), 6.73 (dd, *J* = 12, 1.2 Hz, 1H), 7.27 (s, 1H), 7.34–740 (m, 3H), 7.66 (dd, *J* = 7, 1.6 Hz, 2H); ^13^C-NMR (100 MHz, CDCl_3_) δ: 114.8 (CH), 119.8 (C), 128.2 (CH), 128.7 (CH), 129.3 (CH), 134.2 (CH), 134.9 (C), 144.1 (CH), 170.8 (C); IR (KBr): 1699 cm^-1^; MS (FAB) *m/z*: 253 (M^+^); HRMS (EI) *m/z*: Calcd for C_11_H_10_BrO_2_ (M^+^) 251.9876, found: 252.9857.

***(2Z,4E)-*4-Methyl-5-phenylpenta-2,4-dienoic acid (2d)**

Colorless needles (CH_2_Cl_2_/hexane), mp. 60.0–62.0 °C; ^1^H-NMR (400 MHz, CDCl_3_) δ: 2.14 (s, 3H), 5.80 (d, *J* = 12 Hz, 1H), 6.71 (d, *J* = 12 Hz, 1H), 6.74 (s, 1H), 7.34–7.37 (m, 5H); ^13^C-NMR (100 MHz, CDCl_3_) δ: 17.3 (CH_3_), 117.1 (CH), 127.5 (CH), 128.2 (CH), 129.6 (CH), 134.3 (C), 136.7 (C), 136.8 (CH), 148.4 (CH), 171.5 (C); IR (KBr): 1679, 2970 cm^-1^; MS (EI) *m/z*: 188 (M^+^); HRMS (EI) *m/z*: Calcd for C_12_H_12_O_2_ (M^+^) 188.0837, found: 188.0842.

**(*Z*)-4-((*Z*)-Benzylidene)-5,5-dimethylhex-2-enoic acid (2e)**

Colorless needles (hexane), mp. 72–74 °C; ^1^H-NMR (400 MHz, CDCl_3_) δ: 1.04 (9H, s), 5.86 (1H, d, *J* = 11.6 Hz), 6.53 (1H, s), 7.01 (1H, dd, *J* = 2.0, 11.6 Hz), 7.15–7.26 (5H, m); ^13^C-NMR (150 MHz, CDCl_3_) δ: 31.0 (CH_3_), 36.4 (C), 119.5 (CH), 126.3 (CH), 127.6 (CH), 128.3 (CH), 128.5 (CH), 139.5 (C), 145.5 (C), 150.7 (CH), 172.2 (C); IR (KBr) 1686 cm^-1^; EIMS *m/z* 230 (M^+^), 57 (100%); Anal. calcd for C_15_H_18_O_2_: C, 78.12; H, 7.91, found: C, 78.23; H, 7.88.

**(2*Z*,4*Z*)-4,5-Diphenylpenta-2,4-dienoic acid (2f)**

Colorless needles (hexane), mp. 85–87 °C; ^1^H-NMR (400 MHz, CDCl_3_) δ: 5.83 (1H, d, *J* = 12 Hz), 6.74 (1H, d, *J* = 12 Hz), 6.83 (1H, s), 7.00–7.31 (10H, m); ^13^C-NMR (150 MHz, CDCl_3_) δ: 119.3 (CH), 127.5 (CH), 127.6 (CH), 127.9 (CH), 128.5 (CH), 129.4 (CH), 129.6 (CH), 134.0 (CH), 136.1 (C), 137.5 (C), 137.9 (C), 146.1 (CH), 171.6 (C); IR (KBr) 1686 cm^-1^; FAB-MS *m/z* 250 (M^+^); Anal. calcd for C_17_H_14_O_2_: C, 81.41; H, 5.46, found: C, 81.58; H, 5.64.

**(2*Z*,4*Z*)-4-Benzylidenedec-2-en-5-ynoic acid (2g)**

Colorless powder (hexane), mp. 73–74 °C; ^1^H-NMR (600 MHz, CDCl_3_) δ: 0.92 (3H, t, *J* = 7.8 Hz), 1.44 (2H, tq, *J* = 7.8, 7.8 Hz), 1.88 (2H, tt, *J* = 7.8, 7.8 Hz), 2.43 (2H, t, *J* = 7.8 Hz), 5.86 (1H, d, *J* = 12 Hz), 6.57 (1H, d, *J* = 12 Hz), 6.88 (1H, s), 7.29–7.92 (5H, m); ^13^C-NMR (150 MHz, CDCl_3_) δ: 13.6 (CH_3_), 19.4 (CH_2_), 22.1 (CH_2_), 30.3 (CH_2_), 77.3 (C), 99.8 (C), 117.5 (C), 118.9 (CH), 128.2 (CH), 129.0 (CH), 129.3 (CH), 135.7 (C), 140.9 (CH), 142.3 (CH), 188.9 (C); IR (KBr) 1676 cm^-1^; FAB-MS *m/z* 254 (M^+^), 255 (M+1); HRMS (FAB) *m/z* calcd for C_17_H_19_O_2_: 254.1307, found: 254.1302

**2-3. Synthesis of 4-arylethynyl analogs 2h-j**

**(*E*)-2-Benzylidene-4-phenylbut-3-ynal (3h)**^5,6^

To a solution of α-bromocinnamaldehyde (1.06 g, 5.0 mmol) and phenylacetylene (766 mg, 7.5 mmol) in benzene (25 mL) was added Pd(PPh_3_)_4_ (231 mg, 0.2 mmol), CuI (95 mg, 0.5 mmol) and *^i^*Pr_2_NH (1.4 mL) under argon. After being stirred 1.5 hr, the reaction was diluted by hexane, washed with water and with brine, dried over MgSO_4_, filtered and concentrated *in vacuo.* The crude product was purified by silica gel column chromatography (10% ethyl acetate in hexane) to give 1.00 g (86%) of (*E*)-2-benzylidene-4-phenylbut-3-ynal (**3h**) as brown oil. ^1^H-NMR (400 MHz, CDCl_3_) δ: 7.26-7.41 (6H, m), 7.48 (1H, s), 7.54-7.61 (3H, m), 8.14-8.16 (2H, m), 9.65 (1H, s). The spectral data were consistent with those reported in the literature.^7^

**(*Z*)-4-((*Z*)-Benzylidene)-6-phenylhex-2-en-5-ynoic acid (2h)**

To a solution of (*E*)-2-benzylidene-4-phenylbut-3-ynal (1.00 g, 4.3 mmol) in THF (10 mL) was added Triton B (40% MeOH solution, 2.57 mL, 6.5 mmol) at -78 °C under argon atmosphere. After stirring for 15 min, 2-[bis(2-isopropylphenoxy)phosphoryl] acetate (2.1 g, 5.2 mmol) in THF (10 mL) was added to the mixture. The resulting mixture was stirred for 1 h at -78 °C, and the reaction was quenched with saturated aqueous NH_4_Cl. The mixture was extracted with EtOAc, and the combined organic layer was washed with brine, dried over MgSO_4_, filtered and concentrated *in vacuo*. The crude product was purified by silica gel column chromatography (hexane/ether = 95/5) to give ethyl ester of **2h** (1.35 g, >99% *Z*:*E* = 95:5) as a yellow oil; ^1^H-NMR (400 MHz, CDCl_3_) δ: 1.20 (3H, t, *J* = 7 Hz), 4.13 (2H, q, *J* = 7 Hz), 5.93 (1H, d, *J* = 12 Hz), 6.55 (1H, d, *J* = 12 Hz), 6.95 (1H, s), 7.27-7.52 (8H, m), 7.96 (2H, d, *J* = 8 Hz); ^13^C-NMR (100 MHz, CDCl_3_) δ: 14.0 (CH_3_), 60.5 (CH_2_), 86.7 (C), 96.9 (C), 117.3 (C), 121.0 (CH), 123.1 (C), 128.3 (CH), 128.4 (CH), 128.5 (CH), 129.1 (CH), 131.5 (CH), 135.7 (C), 138.6 (CH), 141.2 (CH), 166.8 (C); IR (neat) 1722, 2204 cm^-1^; MS (EI) *m/z:* 302 (M^+^), 273 (M-Et). HRMS (EI) Calcd for C_21_H_18_O_2_ (M^+^): 302.1307, found: 302.1307.

To a solution of the ester (500 mg, 1.65 mmol) in EtOH (8.0 mL) was added 10% NaOH aq (8.0 mL) at room temperature. After being stirred for 4 h, the mixture was washed with hexane and extracted with water. The combined aqueous layer was acidified with 3M HCl. The mixture was extracted with EtOAc. The organic layer was washed with brine, dried over MgSO_4_, filtered and concentrated *in vacuo*. The crude product was recrystallized from CH_3_CN to give 368 mg (81%) of **2h** as yellow solid**:** Pale yellow crystals (CH_3_CN), mp. 119–121 °C; ^1^H-NMR (400 MHz, CDCl_3_) δ: 5.86 (1H, d, *J* = 12 Hz), 6.62 (1H, dd, *J* = 1.2, 12 Hz), 6.98 (1H, s), 7.32–7.50 (8H, m), 7.96 (2H, d, *J* = 7.6 Hz); ^13^C-NMR (100 MHz, CDCl_3_) δ: 86.3 (C), 97.5 (C), 116.9 (C), 119.4 (CH), 122.9 (C), 128.3 (CH), 128.6 (CH), 129.3 (CH), 129.6 (CH), 131.5 (CH), 135.6 (C), 141.2 (CH), 142.0 (2CH), 143.3 (CH), 171.7 (C); IR (KBr) 1685, 2200 cm^-1^; FAB-MS *m/z* 274 (M^+^); Anal. calcd for C_19_H_14_O_2_: C, 83.07; H, 5.08. found: C, 83.19; H, 5.14.

**(*E*)-2-Benzylidene-4-(4-methoxyphenyl)but-3-ynal (3i)**

To a solution of α-bromocinnamaldehyde (1.06 g, 5.02 mmol) and 1-ethynyl-4-methoxybenzene (991 mg, 7.50 mmol) in benzene (25 mL) was added Pd(PPh_3_)_4_ (231 mg, 0.200 mmol), CuI (95 mg, 0.50 mmol) and *i*-Pr_2_NH (1.4 mL) under argon. After being stirred 4 h, the mixture was diluted by hexane, washed with water and brine, dried over MgSO_4_, filtered and concentrated *in vacuo.* The crude product was purified by silica gel column chromatography (10% ethyl acetate in hexane) to give 1.25 g (95%) of (*E*)-2-benzylidene-4-(4-methoxyphenyl)but-3-ynal as orange oil; ^1^H-NMR (270 MHz, CDCl_3_) δ: 3.85 (3H, s), 6.90-6.93 (2H, m), 7.45-7.57 (5H, m), 8.09-8.20 (2H, m), 9.64 (1H, s).

**Analogue 2i (BMA)**

To a solution of the aldehyde (1.25 g, 4.76 mmol) in THF (15 mL) was added Triton B (40% MeOH solution, 3.0 mL, 7.1 mmol) at −78 °C under argon atmosphere. After stirring for 20 min, 2-[bis(2-isopropylphenoxy)phosphoryl] acetate (2.3 g, 5.7 mmol) in THF (10 mL) was added to the mixture. After the resulting mixture was stirred for 1 h at −78 °C, the reaction was quenched with saturated aqueous NH_4_Cl. The mixture was extracted with EtOAc, and the combined organic layer was washed with brine, dried over MgSO_4_, filtered and concentrated *in vacuo*. The crude product was purified by silica gel column chromatography (hexane/EtOAc= 9/1) to give the ethyl ester (1.45 g, 93% *Z*:*E* = 94:6) as a yellow oil, which was partially separated by column chromatography; ^1^H-NMR (600 MHz, CDCl_3_) δ: 1.20 (3H, t, *J* = 7 Hz), 3.82 (3H, s), 4.13 (2H, q, *J* = 7 Hz), 5.91 (1H, d, *J* = 13 Hz), 6.53 (1H, d, *J* = 13 Hz), 6.88 (2H, d, *J =* 8 Hz), 6.90 (1H, S), 7.30-7.46 (5H, m), 7.95 (2H, d, *J=* 7 Hz). ^13^C-NMR (150 MHz, CDCl_3_) δ: 14.0 (CH_3_), 55.3 (CH_3_), 60.6 (CH_2_), 85.5 (C), 97.1 (C), 114.0 (CH), 115.2 (C), 117.6 (C), 120.9 (CH), 128.2 (CH), 128.9 (CH), 129.4 (CH), 132.9 (CH), 135.9 (C), 138.6 (CH), 140.4 (CH), 159.8 (C), 167.0 (C). IR (neat) 1718, 2201 cm^-1^. MS (FAB, 3NBA) *m/z:* 332 (M^+^), 303 (M-Et), 287 (M-OEt). HRMS (FAB) Calcd for C_21_H_18_O_2_ (M^+^): 332.1412, found: 332.1409.

To a solution of the ester (423 mg, 1.27 mmol) in EtOH (8.0 mL) was added 10% NaOH aq (8.0 mL) at room temperature. After being stirred for 5 h, the mixture was washed with hexane and extracted with water. The combined aqueous layer was acidified with 3M HCl. The mixture was extracted with EtOAc. The organic layer was washed with brine, dried over MgSO_4_, filtered and concentrated *in vacuo*. The crude product was recrystallized from CH_3_CN to give 326 mg (85%) of BMA as pale yellow solid**:** Pale yellow solid (CH_3_CN), mp. 108–110 °C; ^1^H-NMR (600 MHz, CD_3_OD) δ: 3.81 (3H, s), 5.96 (1H, d, *J* = 12.6 Hz), 6.53 (1H, d, *J* = 12.6 Hz), 6.54–7.99 (10H, m); ^13^C-NMR (150 MHz, CD_3_OD) δ: 55.8 (CH_3_), 86.1 (C), 99.2 (C), 115.2 (CH), 116.4 (C), 119.2 (C), 122.2 (CH), 129.4 (CH), 130.1 (CH), 130.4 (CH), 134.0 (CH), 137.4 (C), 138.5 (CH), 141.4 (CH), 161.6 (C), 171.1 (C); IR (KBr) 1692, 2201 cm^-1^; FAB-MS *m/z* 304 (M^+^), 154 (100%); HRMS (FAB) *m/z*: Calcd for C_20_H_16_O_3_ (M^+^): 304.1099, found: 304.1101.

**(*E*)-2-Benzylidene-4-(4-(pyrrolidine-1-carbonyl)phenyl)but-3-ynal (3j)**

To a solution of 4-bromobenzoic acid (1.6 g, 8.0 mmol) in CH_2_Cl_2_ (50 mL) was added oxalyl chloride (0.89 mL, 10 mmol) and DMF (1 drop) at room temperature. After the mixture was stirred for 1 h, *i*Pr_2_NEt (1.82 mL, 10.4 mmol) and pyrrolidine (0.86 mL, 10.4 mmol) was then added to the mixture. After stirred for 19 h at room temperature, water (20 mL) was added. The mixture was extracted with CH_2_Cl_2_, and the combined organic layer was washed with sat. NaHCO_3_ aq and brine, dried over MgSO_4_, filtered, and concentrated *in vacuo*. The crude product was purified by silica gel column chromatography (EtOAc/CHCl_3_ = 3/97) and recrystallization (hexane) to give (4-bromophenyl)(pyrrolidin-1-yl)methanone (1.22 g, 60%) as colorless crystal.

To a solution of (4-bromophenyl)(pyrrolidin-1-yl)methanone (1.22 g, 4.80 mmol) in benzene (25 mL) was added trimetylsilylacetylene (707 mg, 7.20 mmol), Pd(Ph_3_P)_4_ (219 mg, 0.189 mmol), CuI (91 mg, 0.48 mmol) and *i-*Pr_2_NH (1.35 mL) under argon atmosphere. After stirring for 7 h at room temperature, the mixture was diluted with hexane (10 mL), washed with brine, dried over MgSO_4_, filtered and concentrated *in* *vacuo*. The crude mixture was purified by silica gel column chromatography (AcOEt/CHCl_3_ = 5/95) to give the 1-pyrrolidinyl[4-[2-(trimethylsilyl)ethynyl]phenyl]methanone (336 mg, 26%) as brown solid.

To a solution of 1-pyrrolidinyl[4-[2-(trimethylsilyl)ethynyl]phenyl]methanone in MeOH (15 mL) was added pottasium carbonate (856 mg, 6.19 mmol) at room temperature. After stirring for 24 h, water (10 mL) was added. The mixture was extracted with EtOAc. The organic layer was washed with brine, dried over MgSO_4_, filtered and concentrated *in vacuo*. The crude product was purified by silica gel column chromatography (hexane/AcOEt = 6/4) to give (4-ethynylphenyl)(pyrrolidin-1-yl)methanone (145 mg, 59%) as orange oil.

To a solution of α-bromocinnamaldehyde (102 mg, 0.483 mmol) and (4-ethynylphenyl)(pyrrolidin-1-yl)methanone (145 mg, 0.728 mmol) in benzene (5.0 mL) was added Pd(PPh_3_)_4_ (34 mg, 0.029 mmol), CuI (14 mg, 0.074 mmol) and *i*-Pr_2_NH (0.20 mL) under argon atmosphere. After being stirred 10 h, the resulting mixture was diluted with hexane, washed with water and brine, dried over MgSO_4_, filtered and concentrated *in vacuo.* The crude product was purified by silica gel column chromatography (CHCl_3_/EtOAc = 98:2) to give (*E*)-2-benzylidene-4-(4-(pyrrolidine-1-carbonyl)phenyl)but-3-ynal (149 mg, >99%) as yellow oil; ^1^H-NMR (400 MHz, CDCl_3_) δ: 1.89-2.00 (qd, J = 12.6, 6.8 Hz, 4H), 3.44 (t, *J* = 6 Hz, 2H), 3.66 (t, *J* = 7 Hz, 2H), 7.64-7.50 (m, 9H), 8.14 (dd, *J* = 3, 7 Hz, 2H), 9.66 (s, 1H).

**(2*Z*,4*Z*)-4-Benzylidene-7-oxo-7-(pyrrolidin-1-yl)hept-2-en-5-ynoic acid (2j)**

To a solution of (*E*)-2-benzylidene-4-(4-(pyrrolidine-1-carbonyl)phenyl)but-3-ynal (**3j**, 149 mg, 0.588 mmol) in THF (8.0 mL) was added Triton B (40% MeOH solution, 0.35 mL, 0.89 mmol) at −78 °C under argon atmosphere. After stirring for 20 min, 2-[bis(2-isopropylphenoxy)phosphoryl] acetate (286 mg, 0.707 mmol) in THF (8.0 mL) was added to the mixture. After the resulting mixture was stirred for 3 h at −78 °C, the reaction was quenched with saturated aqueous NH_4_Cl. The mixture was extracted with EtOAc, and the combined organic layer was washed with brine, dried over MgSO_4_, filtered and concentrated *in vacuo*. The crude product was purified by silica gel column chromatography (CHCl_3_/EtOAc= 19/1) to give ethyl (Z)-4-((Z)-benzylidene)-6-(4-(pyrrolidine-1-carbonyl)phenyl)hex-2-en-5-ynoate (152 mg, 80%) as a yellow oil; ^1^H-NMR (400 MHz, CDCl_3_) δ: 1.21 (3H, t, *J* = 7 Hz), 1.88-2.01 (4H, m), 3.43 (2H, t, *J* = 7 Hz), 3.66 (2H, t, *J* = 7 Hz), 4.13 (2H, q, *J* = 7 Hz), 5.94 (1H, d, *J* = 11 Hz), 6.56 (1H, d, *J* = 11 Hz), 6.98 (1H, s), 7.33-7.43 (5H, m), 7.49- 7.58 (2H, m), 7.94 (2H, d, *J* = 8 Hz). ^13^C-NMR (150 MHz, CDCl_3_) δ: 14.0 (CH_3_), 26.4 (CH_2_), 26.4 (CH_2_), 46.3 (CH_2_), 49.5 (CH_2_), 60.6 (CH_2_), 88.0 (C), 96.0 (C), 117.2 (C), 121.2 & 121.3 (CH, rotamer), 124.7 (C), 127.3 & 127.4 (CH, rotamer), 128.3 & 128.5 (CH, rotamer), 129.5 (CH), 131.3 & 131.5 (CH, rotamer), 131.4 (CH), 135.7 (C), 137.1 (C), 138.5 (CH), 141.8 & 142.0 (CH, rotamer), 166.8 (C), 168.9 (C). IR (KBr) 1614, 1722, 2374 cm^-1^. FAB-MS *m/z* 400 (M+H), 154 (100%); HRMS (FAB) *m/z*: Calcd for C_26_H_26_NO_3_ (M+H): 400.1913, found: 400.1917.

To a solution of ethyl (Z)-4-((Z)-benzylidene)-6-(4-(pyrrolidine-1-carbonyl)phenyl)hex-2-en-5-ynoate (112 mg, 0.346 mmol) in ethanol (5 mL) was added 10% NaOH aq. (5 mL). After being stirred at room temperature for 3.5 h, the mixture was diluted with hexane and extracted with H_2_O. The aqueous layer was acidified with 3M HCl and the mixture was extracted with EtOAc. The organic layer was washed with brine, dried over MgSO_4_ and concentrated *in vacuo* to give the crude product, which was purified by silica gel column chromatography (CHCl_3_/EtOAc) to yield 94 mg (95%) of **2j:** Yellow powder; mp. 85–105 °C (dec.); ^1^H-NMR (600 MHz, CDCl_3_) δ: 1.81–1.90 (4H, m), 3.35 (2H, t, *J* = 6 Hz), 3.64 (2H, t, *J* = 6 Hz), 5.96 (1H, d, *J* = 12 Hz), 6.56 (1H, d, *J* = 12 Hz), 6.95 (1H, s), 7.30–7.39 (5H, m), 7.49 (2H, d, *J* = 8 Hz), 7.94 (2H, d, *J* = 8 Hz); ^13^C-NMR (150 MHz, CDCl_3_) δ: 24.3 (CH_2_), 26.3 (CH_2_), 46.5 (CH_2_), 49.8 (CH_2_), 87.8 (C), 96.8 (C), 117.4 (C), 121.3 (CH), 124.9 (C), 127.3 (CH), 128.3 (CH), 129.3 (CH), 129.5 (CH), 131.4 (CH), 135.7 (C), 135.9 (C), 137.8 (CH), 141.8 (CH), 166.8 (C), 169.3 (C); IR (KBr) 1595, 1701, 2372 cm^-1^; FAB-MS *m/z* 372 (M+H), 154 (100%); HRMS (FAB) *m/z*: Calcd for C_24_H_22_NO_3_ (M+H): 372.1601, found: 372.1600.

**2-4. Synthesis of analogues 2k-p**

**General procedure for synthesis of analogues 2k-p: Synthesis of 2k**

**(*Z*)-Ethyl 2-bromo-3-(2-methoxy)phenylacrylate**

To a solution of 3-methoxybenzaldehyde (1.0 g, 7.4 mmol) and ethyl bromoacetate (1.35 g, 8.0 mmol) in CH_2_Cl_2_ (20 mL) under argon atmosphere was added TiCl_4_ (1.67 g, 8.8 mmol) in drops over a period of 10 minutes. The mixture was then stirred at rt for 30 min. Et_3_N (1.5 g, 7.4 mmol) was added to the solution dropwise over a period of 10 minutes while maintaining the reaction temperature below 30 °C. After the brown mixture was stirred at rt for 9 h, the mixture was diluted with CH_2_Cl_2_ (10 mL) and quenched with HCl (to pH = 1), washed with water, and extracted with CH_2_Cl_2_. The organic layer was washed with brine, dried over Na_2_SO_4_, and filtered. After evaporation, 2.78 g of yellow oil product was obtained, and was purified by column chromatography to yield of pale yellow oil (0.76 g, 69%, *E*:*Z* = 3:100).

A pale yellow oil : ^1^H-NMR (CDCl_3_, 600 MHz) δ: 1.38 (t, *J* = 7 Hz, 3H), 3.87 (s, 3H), 4.35 (q, *J* = 7 Hz, 2H), 6.91 (d, *J* = 8 Hz, 1H), 7.01 (t, *J* = 8 Hz 1H), 7.39 (t, *J* = 8 Hz, 1H), 8.04 (d, *J* = 8 Hz, 1H), 8.45 (s, 1H); ^13^C-NMR (CDCl_3_, 150 MHz) δ: 14.2 (CH_3_), 55.6 (CH_3_), 62.6 (CH_2_), 110.5 (CH), 113.8 (C), 120.0 (CH), 123.0 (C), 130.0 (CH), 131.4 (CH), 136.6 (CH), 157.8 (C), 163.4 (C); IR (neat) 1714, 2980 cm^-1^; MS (EI) *m/z:* 284 (M^+^), 286 (M+2), 205 (M-Br), HRMS (EI) calcd for C_12_H_13_O_3_Br 284.0048, found 284.0047.

**(*Z*)-Ethyl 2-bromo-3-(2-methoxy)phenyl-2-en-1-ol**

To a solution of (*Z*)-ethyl 2-bromo-3-(3-methoxyphenyl)acrylate (1.4 g, 4.9 mmol) in THF (25 mL) was added dropwise DIBAL (1M in toluene,12.8 mL, 12.8 mmol) and the mixture was stirred at −78 °C for 3 h. Saturated potassium sodium (+)–tartrate solution was added and the mixture was stirred for 6 h. Then, the mixture was washed with water and extracted with CH_2_Cl_2_. The organic layer was washed with brine, dried over Na_2_SO_4_, and filtered. After evaporation, 1.24 g of the crude product was obtained, and then purified by column chromatography to yield the tile compound as colorless oil (865 mg, 54%, *E*:*Z*= 2:100); ^1^H-NMR (CDCl_3_, 400 MHz) δ: 2.11 (t, *J* = 7 Hz 1H), 3.84 (s, 3H), 4.44 (d, *J* = 7 Hz, 2H), 6.89 (d, *J* = 9 Hz, 1H), 6.98 (t, *J* = 7 Hz 1H), 7.21 (s, 1H), 7.31 (t, *J* = 7 Hz, 1H), 7.78 (d, *J* = 8 Hz, 1H); ^13^C-NMR (CDCl_3_, 150 MHz) δ: 55.4 (CH_3_), 69.3 (CH_2_), 110.3 (CH_2_), 120.0 (CH_2_), 123.6 (CH_2_), 124.0 (C), 126.2 (C), 129.4 (CH), 129.7 (CH), 156.9 (C); IR (neat) 1599, 2936, 3345 cm^-1^; MS (EI) *m/z*: 242 (M^+^), 244 (M+2), 163 (M-Br), HRMS (EI) *m/z:* calcd for C_10_H_11_O_2_Br, 241.9942, found 241.9945.

**(*Z*)-2-Bromo-3-(2-methoxy)phenyl-2-propenal**

To a solution of (*Z*)-2-bromo-3-(2-methoxy)phenylprop-2-en-1-ol (865 mg, 3.56 mmol) in CH_2_Cl_2_ (30 ml) was added MnO_2_ (6.2 g, 72 mmol) at room temperature. After stirring for 6 h, the mixture was filtered through celite. The filtrate was evaporated *in* *vacuo* to yield the title compound (700 mg, 82%) as pale yellow oil; ^1^H-NMR (CDCl_3_, 600 MHz) δ: 3.92 (s, 3H), 6.96 (d, *J* = 9 Hz, 1H), 7.07 (t, *J* = 8 Hz, 1H), 7.47 (t, *J* = 8 Hz, 1H), 8.32 (s, 1H), 8.36 (d, *J* = 8, 1H), 9.37 (s, 1H); ^13^C-NMR (CDCl_3_, 150 MHz) δ: 55.7 (CH3), 110.8 (CH), 120.3 (CH), 122.0 (C), 124.6 (C), 130.0 (CH), 133.0 (CH), 144.3 (CH), 158.1 (C), 187.3 (CH); IR (neat) 1682, 1697 cm^-1^; MS (EI) m/z: 240 (M^+^). 242 (M+2), 161 (M-Br); HRMS (EI) m/z: calcd for C_10_H_9_O_2_Br, 239.9786, found 239.9787.

**(*E*)-2-(2-Methoxy)benzylidene-4-phenylbut-3-ynal**

Under argon atmosphere, to a solution of (*Z*)-2-bromo-3-(2-methoxy)phenyl-2-propenal (200 mg, 0.830 mmol) and phenylacetylene (127 mg, 1.24 mmol) in benzene (20 mL), was added tetrakis(tripheylphosphine)palladium (38 mg, 0.033 mmol), copper (I) iodide (16 mg, 0.083 mmol) and *i-*Pr_2_NEt (0.29 ml, 1.7 mmol) at room temeprature. After stirring for 22 h, the mixture was washed with water, extracted with AcOEt. The combined organic layer was washed with brine, dried over Na_2_SO_4_, filtered and concentrated to afford 423 mg of brown oil, which was chromatographed over silica gel (EtOAc/hexane = 5/95) to yield 87 mg (40%) of (*E*)-2-(2-methoxy)benzylidene-4-phenylbut-3-ynal as a yellow oil; ^1^H-NMR (CDCl_3_, 600 MHz) δ: 3.93 (s, 3H), 6.97 (d, *J* = 8 Hz, 1H), 7.06 (t, *J* = 8 Hz, 1H), 7.36-7.38 (m, 3H), 7.46 (t, *J* = 8 Hz, 1H), 7.56-7.58 (m, 2H), 8.09 (s, 1H), 8.72 (d, *J* = 8 Hz, 1H), 9.66 (s, 1H); ^13^C-NMR (CDCl_3_, 150 MHz) δ: 55.7 (CH3), 83.3 (C), 100.5 (C), 110.8 (CH), 120.5 (CH), 122.3 (C), 122.7 (C), 123.2 (C), 128.4 (CH), 128.9 (CH), 129.7 (CH), 131.8 (CH), 133.2 (CH), 146.0 (CH), 158.3 (C), 191.5 (CH); IR (neat) 1691 cm^-1^; MS (EI) m/z: 262 (M^+^), 231 (M-CH_3_), HRMS (EI) *m/z:* calcd for C_12_H_18_O_2_, 262.0994, found 262.0993.

**Ethyl (*Z)*-4-(((*Z*)-2-methoxy)benzylidene)-6-phenylhex-2-en-5-ynoate**

Under Ar atmosphere, to a solution of Ethyl 2-[bis(2-isopropylphenoxy)phosphoryl]acetate (300 mg, 0.742 mmol) in THF (7.0 mL) was added dropwise Triton B (0.37 mL, 0.81 mmol, 40% MeOH) at −78 °C. After 15 min, a solution of (*E*)-2-benzylidene-4-(2-methoxy)phenylbut-3-ynal (162 mg, 0.618 mmol) in THF (7.0 mL) was added dropwise. After being stirred for 3 h at −78 °C, the reaction was quenched with sat. NH_4_Cl. The mixture was extracted with EtOAc. The combined organic layer was washed with brine, dried over by Na_2_SO_4_, filtered and concentrated *in vacuo* to yield the crude product, which was purified by column chromatography (hexane/EtOAc = 9/1) to provide 126 mg (61%) of the title compound as a yellow oil; ^1^H-NMR (CDCl_3_, 600 MHz) δ: 1.20 (t, *J* = 7 Hz, 3H), 3.86 (s, 3H), 4.13 (q, *J* = 7 Hz, 2H), 5.90 (d, *J* = 12 Hz, 1H), 6.59 (d, *J* = 13 Hz, 1H), 6.90 (d, *J* = 8 Hz, 1H), 7.00 (t, *J* = 8 Hz, 1H), 7.31-7.33 (m, 4H), 7.36 (s, 1H), 7.46-7.47 (m, 2H), 8.48 (dd, *J*^1^ = 7.9 Hz*,* 1.7 Hz, 1H); ^13^C-NMR (CDCl_3_, 150 MHz) δ : 14.0 (CH3), 55.5 (CH3), 60.5 (CH2), 87.0 (C), 96.2 (C), 110.3 (CH), 117.0 (C), 120.1 (CH), 120.4 (CH), 123.3 (C), 124.7 (C), 128.2 (CH), 128.3 (CH), 129.1 (CH), 130.4 (CH), 131.4 (CH), 135.9 (CH), 138.9 (CH), 157.5(C), 167.0 (C); IR (neat) 1714, 2202 cm^-1^; MS (EI) *m/z*: 332 (M^+^), 303 (M-Et), HRMS (EI) *m/z:* calcd for C_22_H_20_O_3_332.1412, found 332.1412.

**(*Z*)-4-((*Z*)-(2-Methoxy)benzylidene)-6-phenylhex-2-en-5-ynoic acid (2k)**

To a solution of ethyl (*Z*)-((*Z*)-benzylidene)-6-(4-methoxy)phenylhex-2-en-5-ynoate (126 mg, 0.379 mmol) in EtOH (10 mL) was added 10%NaOH aq. (5.3 mL, 13 mmol). After being stirred for 6.5 h at room temperature, the mixture was added into hexane, extracted with H_2_O. The aqueous layer was acidified with HCl. The mixture was extracted with EtOAc and the organic layer was washed with brine, dried over by Na_2_SO_4_, filtered, and concentrated *in vacuo* to yield the title compound (116 mg, >99%) as a pale yellow viscous oil; ^1^H-NMR (CDCl_3_, 400 MHz) δ: 3.86 (s, 3H), 5.87 (d, *J* = 12 Hz, 1H), 6.67 (d, *J* = 12 Hz, 1H), 6.90 (d, *J* = 8 Hz, 1H), 7.00 (t, *J* = 8 Hz, 1H), 7.29-7.32 (m, 4H), 7.42 (s, 1H), 7.45 (d, *J* = 3 Hz, 2H), 8.50 (d, *J* = 8 Hz, 1H); ^13^C-NMR (CDCl_3_, 100 MHz) δ: 55.5 (CH_3_), 86.6 (C), 96.8 (C), 110.4 (CH), 116.7 (C), 118.9 (CH), 120.1 (CH) 123.1 (C), 124.6 (C), 128.2 (CH), 128.3 (CH), 129.2 (CH), 130.7 (CH), 131.4 (CH), 136.8 (CH), 141.4 (CH), 157.7 (C), 171.5 (C); IR (KBr) 1693 cm^-1^; MS (EI) *m/z*: 304 (M^+^), 289 (M-Me), HRMS (EI) calcd for C_20_H_16_O_3_ m/z: 304.1099, found 304.1098.

**Synthesis of 2l**

**(*Z*)-Ethyl 2-bromo-3-(3-methoxy)phenylacrylate**

66%, *E*:*Z* = 1:10. ^1^H-NMR (CDCl_3_, 600 MHz) δ: 1.39 (t, *J* = 7 Hz, 3H), 3.85 (s, 3H), 4.35 (q, *J* = 7 Hz, 2H), 6.98 (dd, *J* = 8, 3 Hz, 1H), 7.34 (t, *J* = 8 Hz 1H), 7.38 (d, *J* = 8 Hz, 1H), 7.46 (d, *J* = 2 Hz, 1H), 8.19 (s, 1H); ^13^C-NMR (CDCl_3_, 150 MHz) δ: 14.2 (CH_3_), 55.3 (CH_3_), 62.8 (CH_2_), 113.3 (C), 115.0 (CH), 116.2 (CH), 123.1 (CH), 129.4 (CH), 134.9 (C), 140.6 (CH), 159.4 (C), 163.3 (C); IR (neat) 1714, 2980 cm^-1^; MS (EI) m/z: 284 (M^+^), 286 (M+2), 205 (M-Br). HRMS (EI) calcd for C_12_H_13_O_3_Br, m/z: 284.0048, found 284.0048.

**(*Z*)-2-Bromo-3-(3-methoxyphenyl)prop-en-1-ol**^8^

^1^H-NMR (CDCl_3_, 400M) δ: 2.14 (br, 1H), 3.83 (s, 3H), 4.42 (d, *J* = 6 Hz, 2H), 6.87 (d, *J* = 7 Hz, 1H), 7.07 (s, 1H), 7.16 (d, *J* = 7 Hz, 1H), 7.22 (s, 1H), 7.29 (t, *J* = 7 Hz, 1H).

**(*Z*)-2-Bromo-3-(3-methoxyphenyl)propenal**^9^

^1^H-NMR (CDCl_3_, 600M) δ: 3.87 (s, 3H), 7.07 (d, *J* = 7 Hz, 1H), 7.40 (dd, J = 7, 7 Hz, 1H), 7.51 (d ,J = 7 Hz, 1H), 7.64 (s, 1H), 7.88 (s, 1H), 9.35 (s, 1H). ^13^C-NMR (CDC_3_, 150 MHz) δ: 55.4 (CH_3_), 115.4 (CH), 117.6 (CH), 123.9 (CH), 124.4 (C), 129.7 (CH), 134.1 (C), 149.0 (CH), 159.6 (C), 187.0 (CH).

**(*E*)-2-(3-Methoxy)benzylidene-4-phenylbut-3-ynal**

84%, a dark yellow oil. ^1^H-NMR (CDCl_3_, 600 MHz) δ: 3.83 (s, 3H), 7.04 (d, *J* = 8 Hz, 1H), 7.38-7.41 (m, 4H), 7.52 (s, 1H), 7.60-7.61 (m, 3H), 7.87 (s, 1H), 9.65 (s, 1H); ^13^C-NMR (CDCl_3_, 150 MHz) δ: 55.3 (CH_3_), 83.22 (C), 101.17 (C), 114.6 (CH), 118.1 (CH), 122.4 (C), 122.7 (C), 123.8 (CH), 128.4 (CH), 129.1 (CH), 129.7 (CH), 131.8 (CH), 135.3 (C), 151.2 (CH), 159.7 (C), 190.9 (CH); IR (neat) 1693, 2204, 2833 cm^-1^; MS (EI) *m/z*: 262 (M^+^), 247 (M-Me), HRMS (EI) *m/z*: calcd for C_18_H_14_O_2_, 262.0994, found 262.0994.

**Ethyl (*Z*)-4-((Z)-(3-methoxy)benzylidene)-6-phenylhex-2-en-5-ynoate**

87%, a yellow oil. ^1^H-NMR (CDCl_3_, 600 MHz) δ: 1.20 (t, *J* = 7.2 Hz, 3H), 3.78 (s, 3H), 4.12 (q, *J* = 7 Hz, 2H), 5.93 (d, *J* = 12 Hz, 1H), 6.54 (d, *J* = 11 Hz, 1H), 6.89 (dd, *J* = 8.2, 2.7 Hz, 1H), 6.93 (s, 1H), 7.29 (t, *J* = 8 Hz, 1H), 7.34-7.35 (m, 3H), 7.43 (d, *J* = 8 Hz, 1H), 7.51-7.52 (m, 2H), 7.68 (s, 1H); ^13^C-NMR (CDCl_3_, 150 MHz) δ: 14.0 (CH_3_), 55.2 (CH_3_), 60.5 (CH_2_), 87.0 (C), 97.2 (C), 113.7 (CH), 115.0 (CH), 117.4 (C), 121.1 (CH), 122.5 (CH), 123.0 (C), 128.3 (CH), 128.6 (CH), 129.2 (CH), 131.4 (CH), 136.9 (C), 138.6 (CH), 141.1 (CH), 159.4 (C), 166.8 (C); IR (neat) 1722, 2980 cm^-1^; MS (EI) m/z: 332 (M+), 303 (M-Et). HRMS (EI) *m/z*: calcd for C_22_H_20_O_3_ 332.1412, found 332.1412.

**(*Z*)-4-((Z)-(3-methoxy)benzylidene)-6-phenylhex-2-en-5-ynoic acid (2l)**

To a solution of ethyl (*Z*)-((*Z*)-benzylidene)-6-(4-methoxy)phenylhex-2-en-5-ynoate (152 mg, 0.46 mmol) in EtOH (10 mL) was added 10% NaOH aq. (2.8 mL, 6.9 mmol) into the mixture and were stirred for 7 h at room temperature. When the reaction was completed, the mixture was added into hexane, extracted with H_2_O, acidified with HCl (to pH = 1), then extracted with EtOAc, and the organic layer was washed by brine, dried over Na_2_SO_4_, filtered and concentrated in vacuo to yield the product (112 mg, 81%) as yellow solid. mp 105.5-106.5 °C (toluene). ^1^H-NMR (CDCl_3_, 600 MHz) δ:3.78 (s, 3H), 5.88 (d, *J* = 12 Hz, 1H),6.62 (d, *J* = 12 Hz, 1H),6.91 (d, *J* = 8 Hz, 1H), 6.95 (s, 1H), 7.29 (t, *J* = 8 Hz, 1H), 7.33-7.34 (m, 3H), 7.43 (d, *J* = 8 Hz, 1H), 7.49-7.50 (m, 2H), 7.69 (s, 1H); ^13^C-NMR (CDCl_3_, 150 MHz) δ: 55.2 (CH3), 86.3 (C), 97.9 (C), 113.7 (CH), 115.8 (CH), 117.0 (C), 119.6 (CH), 122.7 (CH), 122.8 (C), 128.3 (CH), 128.6 (CH), 129.2 (CH), 131.4 (CH), 136.8 (C), 141.0 (CH), 141.9 (CH), 159.4 (C), 171.9 (C); IR (KBr) 1687, 2958 cm^-1^; HRMS (EI) calcd for C_20_H_16_O_3_, 304.1099, found 304.1098.

**Synthesis of 2m**

**Ethyl (*Z*)-2-bromo-3-(4-methoxyphenyl)propenoic acid**^10^

73%, *Z : E* = 98:2, pale yellow oil, ^1^H-NMR (CDCl_3_, 400 MHz) δ: 1.38 (t, *J* = 7 Hz, 3H), 3.82 (s, 3H), 4.34 (q, *J* = 7 Hz, 2H), 6.95 (d, *J* = 8 Hz, 2H), 7.89 (d *J* = 8 Hz, 2H), 8.17 (s, 1H); IR (neat) 1600, 1714, 2980 cm^-1^; MS (EI) m/z: 284 (M^+^), 286 (M+2), 205 (M-Br).

**(*Z*)-2-Bromo-3-(4-methoxyphenyl)prop-2-en-1-ol**^8^

54%, colorless solid, ^1^H-NMR (CDCl_3_, 600 MHz) δ: 2.23 (br, 1H), 3.82 (s, 3H), 4.39 (s, 2H), 6.91 (d, *J* = 8 Hz, 2H), 7.61 (d *J* = 8 Hz, 2H).

**(*Z*)-2-Bromo-3-(4-methoxyphenyl)acrylaldehyde**^11^

80%, pale yellow crystal, ^1^H-NMR (CDCl_3_, 400 MHz) δ: 3.89 (s, 3H), 7.01 (d, *J* = 8 Hz, 2H), 8.04 (d *J* = 8 Hz, 2H), 9.30 (1H, s).

**(*E*)-2-(4-Methoxybenzylidene)-4-phenylbut-3-ynal**^12^

73%, yellow oil, ^1^H-NMR (CDCl_3_, 400 MHz) δ: 3.89 (s, 3H), 6.99 (d, *J* = 8 Hz, 2H), 7.39 (3H, m), 7.48 (1H, s), 7.60 (2H, m), 8.14 (d, *J* = 8 Hz, 2H), 9.60 (1H, s).

**Ethyl (*Z*)-4-((Z)-(4-methoxy)benzylidene)-6-phenylhex-2-en-5-ynoate**

78%, yellow oil as isomers mixture (*Z:E* = 100:5). ^1^H-NMR (CDCl_3_, 600 MHz) δ: 1.20 (t, *J* = 7 Hz, 3H), 3.83 (s, 3H), 4.12 (q, *J* = 7 Hz, 2H), 5.87 (d, *J* = 12 Hz, 1H), 6.51 (d, *J* = 12 Hz, 1H), 6.90-6.92 (m, 3H), 7.33-7.38 (m, 3H), 7.51-7.52 (m, 2H), 7.94 (d, *J* = 9 Hz, 2H); ^13^C-NMR (CDCl_3_, 150 MHz) δ: 14.0 (CH_3_), 55.3 (CH_3_), 60.5 (CH_2_), 87.0 (C), 96.6 (C), 113.7 (CH), 114.9 (C), 120.0 (CH), 123.2 (C), 128.3 (CH), 128.4 (CH), 128.6 (C), 131.1 (CH), 131.4 (CH), 139.0 (CH), 141.2 (CH), 160.3 (C), 167.0 (C); IR (neat) 1714, 2980 cm^-1^; MS (EI) *m/z:* 332 (M^+^), 303 (M-Et). HRMS (EI) *m/z*: calcd for C_22_H_20_O_3_, 332.1412, found 332.1412.

**(*Z*)-4-((Z)-(4-Methoxy)benzylidene)-6-phenylhex-2-en-5-ynoic acid (2m)**

Recrystallization from CHCl_3_/hexane yielded a pure product as plates (mp. 116-118 °C) ^1^H-NMR (CDCl_3_, 400 MHz) δ: 3.84 (s, 3H), 5.80 (d, *J* = 12.6 Hz, 1H), 6.59 (d, *J* = 12.6 Hz, 1H), 6.91 (d, *J* = 8.7 Hz, 2H), 6.94 (s, 1H), 7.32-7.34 (m, 3H), 7.48-7.50 (m, 2H), 7.95 (d, *J* = 8.7 Hz, 2H); ^13^C-NMR (CDCl_3_, 100 MHz) δ: 55.3 (CH_3_), 86.7 (C), 97.2 (C), 113.8 (CH), 114.6 (C), 118.3 (CH), 123.1 (C), 128.3 (CH), 128.4 (CH), 128.5 (C), 131.3 (CH), 131.4 (CH), 141.5 (CH), 142.1 (CH), 160.5 (C), 170.7 (C); IR (KBr) 1600, 1686, 2202, 2960 cm^-1^; MS (EI) *m/z:* 304 (M^+^), 289 (M-Me), 273 (M-MeO). HRMS (EI) *m/z:* calcd for C_20_H_16_O_3_ 304.1099, found 304.1099.

**Synthesis of 2n**

**(*Z*)-Ethyl 2-bromo-3-(2-fluoro)phenylpropenoate**

56%; Colorless oil, *E*:*Z* = 1:20. ^1^H-NMR (CDCl_3_, 600 MHz) δ: 1.39 (t, *J* = 7 Hz, 3H), 4.37 (q, *J* = 7 Hz, 2H), 7.12 (dd, *J* = 9, 9 Hz, 1H), 7.22 (dd, *J* = 8, 8 Hz, 1H), 7.39-7.43 (m, 1H), 8.12 (dd, *J* = 8, 8 Hz, 1H), 8.35 (s, 1H); ^13^C-NMR (CDCl_3_, 150 MHz) δ: 14.1 (CH_3_), 62.9 (CH_2_), 115.5 (CH, d, *J*^2^_C,F_ = 22 Hz), 116.0 (C), 122.1 (C, *J*^2^_C,F_ = 11 Hz), 123.7 (CH, d, *J*^3^_C,F_ = 4.4 Hz), 129.8 (CH), 131.7 (CH, d, *J*^3^_C,F_ = 8.7 Hz), 133.3 (CH, d, *J*^3^_C,F_ = 5.8 Hz), 160.6 (C, d, *J*^1^_C,F_ = 250 Hz), 162.8 (C); IR (neat) 1716, 1726, 2983 cm^-1^; MS (EI) *m/z*: 272 (M^+^), 274 (M+2), 193 (M-Br); HRMS (EI) calcd for C_11_H_10_O_2_BrF *m/z*: 271.9848, found 271.9847.

**(*Z*)-2-Bromo-3-(2-fluoro)phenyl-2-en-1-ol**

94%; Colorless oil, single *Z* isomer, ^1^H-NMR (CDCl_3_, 400 MHz) δ: 2.17 (br, 1H), 4.45 (d, *J* = 6 Hz, 2H), 7.07 (dd, *J* = 9, 9 Hz, 1H), 7.16-7.18 (m, 2H), 7.31 (m, 1H), 7.84 (t, *J* = 8 Hz, 1H); ^13^C-NMR (CDCl_3_, 150 MHz) δ: 68.7 (CH_2_), 115.1 (CH, d, *J*^2^_C,F_ = 22 Hz), 120.4 (C, d, *J*^3^_C,F_ = 4 Hz), 122.9 (CH, d, *J*^2^_C,F_ = 13 Hz), 123.4 (CH, d, *J*^4^_C,F_ = 3 Hz), 128.1 (C), 129.6 (CH, d, *J*^3^_C,F_ = 9 Hz), 129.8 (CH), 160.0 (C, d, *J*^1^_C,F_ = 247 Hz); IR (neat) 2920, 3346 cm^-1^; MS (EI) *m/z:* 230 (M^+^), 232 (M+2), 151 (M-Br); HRMS (EI) *m/z:* calcd for C_9_H_8_OBrF 229.9743, found 229.9744.

**(*Z*)-2-Bromo-3-(2-fluoro)phenyl-2-propenal**

84%; Pale yellow crystal. ^1^H-NMR (CDCl_3_, 600 MHz) δ: 7.18 (dd, *J* = 9, 9 Hz, 1H), 7.28 (dd, *J* = 7, 7 Hz, 1H), 7.50 (m, 1H), 8.16 (s, 1H), 8.43 (dd, *J* = 7, 8 Hz, 1H), 9.38 (s, 1H); ^13^C-NMR (CDCl_3_, 150 MHz) δ: 115.8 (CH, d, *J*^2^_C,F_ = 22 Hz), 121.2 (C, d, *J*^2^_C,F_ = 11 Hz), 124.1 (CH, d, *J*^4^_C,F_ = 4 Hz), 126.4 (CH), 129.9 (CH) 133.3 (CH, d, *J*^3^_C,F_ = 10 Hz), 140.6 (d, *J*^3^_C,F_ = 7.2 Hz), 160.9 (s, *J*^1^_C,F_ = 253 Hz), 186.7 (s); IR (neat) 1701 cm^-1^; MS (EI) m/z: 228 (M^+^), 230 (M+2), 149 (M-Br); HRMS (EI) calcd for C_9_H_6_OBrF m/z: 227.9586, found 227.9586.

**(*E*)-2-(2-fluoro)benzylidene-4-phenylbut-3-ynal**

80%; Yellow oil. ^1^H-NMR (CDCl_3_, 600 MHz) δ: 7.17 (dd, *J* = 9, 9 Hz, 1H), 7.27 (dd, *J* = 7, 7 Hz, 1H), 7.38-7.39 (m, 3H), 7.47 (dd, *J* = 7, 14 Hz, 1H), 7.57-7.58 (m, 2H), 7.85 (s, 1H), 8.75 (t, *J* = 7 Hz, 1H), 9.67 (s, 1H); ^13^C-NMR (CDCl_3_, 150 MHz) δ: 83.0 (C), 101.6 (C), 115.7 (CH, d, *J*^2^_C,F_ = 22 Hz), 122.3 (C), 122.4 (C, d, *J*^2^_C,F_ = 10 Hz), 123.9 (CH), 124.3 (CH, d, *J*^4^_C,F_ = 4 Hz), 128.5 (CH), 129.2 (CH), 129.5 (CH), 131.8 (CH), 133.3 (CH, d, *J*^3^_C,F_ = 9 Hz), 141.8 (CH, d, *J*^3^_C,F_ = 7 Hz), 161.1 (C, d, *J*^1^_C,F_ = 253 Hz), 190.7 (C); IR (neat) 1699, 2260 cm^-1^; MS (EI) *m/z*: 250 (M^+^); HRMS (EI) calcd for C_17_H_11_OF *m/z*: 250.0794, found 250.0794.

**Ethyl (*Z*)-4-((Z)-(2-fluoro)benzylidene)-6-phenylhex-2-en-5-ynoate**

87%; Yellow oil, 2*E*:*2Z* = 6:100, ^1^H-NMR (CDCl_3_, 400 MHz) δ: 1.21 (t, *J* = 7 Hz, 3H), 4.14 (q, *J* = 7 Hz, 2H), 5.96 (d, *J* = 12 Hz, 1H), 6.58 (d, *J* = 12 Hz, 1H), 7.08 (t, *J* = 9 Hz, 1H), 7.17-7.19 (m, 2H), 7.30-7.35 (m, 4H), 7.47-7.49 (m, 2H), 8.54 (t, *J* = 8 Hz, 1H); ^13^C-NMR (CDCl_3_, 100 MHz) δ : 14.0 (CH_3_), 60.6 (CH_2_), 86.3 (C), 97.2 (C), 115.2 (CH, d, *J*^2^_C,F_ = 23 Hz), 118.8 (C), 121.6 (CH), 122.89 (C), 123.7 (CH), 123.8 (C, d, *J*^2^_C,F_ = 11 Hz), 128.4 (CH), 128.7 (CH), 129.0 (CH), 130.6 (CH, d, *J*^3^_C,F_ = 8.6 Hz), 131.5 (CH), 132.0 (CH, d, *J*^3^_C,F_ = 7 Hz), 138.3 (CH), 160.7 (C, d, *J*^1^_C,F_ = 250 Hz), 166.7 (C); IR (neat) 1728, 2250, 3019 cm^-1^; MS (EI) *m/z*: 320 (M^+^), 291 (M-Et); HRMS (EI) calcd for C_21_H_17_O_2_ *m/z*: 320.1213, found 320.1214.

**(*Z*)-4-((Z)-(2-Fluoro)benzylidene)-6-phenylhex-2-en-5-ynoic acid (2n)**

91%; Colorless crystal, mp. 116-119 °C (CH_2_Cl_2_). ^1^H-NMR (CDCl_3_, 400 MHz) δ: 5.92 (d, *J* = 13 Hz, 1H), 6.65 (d, *J* = 13 Hz, 1H), 7.09 (m, 1H), 7.21 (m, 1H), 7.23 (s, 1H), 7.31-7.37 (m, 5H), 7.48 (m, 2H), 8.57 (dd, *J* = 8, 8 Hz, 1H); ^13^C-NMR (CDCl_3_, 150 MHz) δ : 85.9 (C), 97.8 (C), 115.3 (CH, d, *J*^2^_C,F_ = 22 Hz), 118.4 (C), 120.1 (CH, d, *J*^3^_C,F_ = 9 Hz), 122.7 (C), 123.6 (C), 123.7 (CH), 128.3 (CH), 128.7 (CH), 129.1 (CH), 130.8 (CH), 131.5 (CH), 132.8 (CH, d, *J*^3^_C,F_ = 7 Hz), 140.7 (CH), 160.7 (C, d, *J*^1^_C,F_ = 250 Hz), 171.9 (C); IR (neat) 1688, 2202, 3061 cm^-1^; MS (EI) *m/z*: 292 (M^+^), 273 (M-F), 247 (M-CO_2_H), HRMS (EI) *m/z:* calcd for C_19_H_13_O_2_F 292.0900, found 292.0902.

**Synthesis of 2o**

**(*Z*)-Ethyl 2-bromo-3-(3-fluoro)phenylacrylate**

71%. *E*:*Z* = 5:100, pale yellow oil. ^1^H-NMR (CDCl_3_, 400 MHz) δ:1.39 (t, *J* = 7 Hz, 3H), 4.36 (q, *J* = 7 Hz, 2H), 7.13 (t, *J* = 9, 2 Hz, 1H), 7.39 (dd, *J* = 15 Hz, 8 Hz, 1H), 7.54 (d, *J* = 8, 1H), 7.66 (d, *J* = 11, 1H), 8.17 (s, 1H); ^13^C-NMR (CDCl_3_, 100 MHz) δ: 14.2 (CH_3_), 62.9 (CH_2_), 114.6 (C), 116.5 (CH, d, ^2^*J*_C,F_ = 23 Hz), 117.0 (CH, d, ^2^*J*_C,F_ = 22 Hz), 126.3 (CH), 129.9 (CH, d, ^3^*J*_C,F_ = 8 Hz), 135.8 (C), 139.4 (CH), 162.8 (C, d, ^1^*J*_C,F_ = 248 Hz), 163.0 (C); IR (neat) 1714, 1728, 2983 cm^-1^; MS (EI) m/z: 272 (M^+^), 274 (M+2), 193 (M-Br), HRMS (EI) *m/z:* calcd for C_11_H_10_O_2_BrF 271.9848, found 271.9848.

**(*Z*)-2-Bromo-3-(3-fluoro)phenyl-2-en-1-ol**

94%, *Z : E* = 100 : 5, white solid: ^1^H-NMR (CDCl_3_, 400 MHz) δ: 2.26 (t, *J* = 6 Hz, 1H), 4.42 (d, *J* = 6 Hz, 2H), 7.00-7.04 (m, 1H), 7.07 (s, 1H), 7.29-7.32 (m, 2H), 7.40 (d, *J* = 10 Hz, 1H); ^13^C-NMR (CDCl_3_, 100 MHz) δ: 69.2 (CH_2_), 115.1 (CH, d, *J*^2^_C,F_ = 21 Hz), 115.5 (CH, d, *J*^2^_C,F_ = 22 Hz), 124.9 (CH, d, *J*^4^_C,F_ = 3 Hz), 126.5 (CH), 129.6 (CH, d, *J*^3^_C,F_ = 9 Hz), 137.0 (C, d, *J*^4^_C,F_ = 8 Hz), 162.5 (C, d, *J*^1^_C,F_ = 247 Hz); IR (neat) 1584, 2918, 3346 cm^-1^; MS (EI) *m/z*: 230 (M^+^), 232 (M+2), 151 (M-Br); HRMS (EI) *m/z*: calcd for C_9_H_8_OBrF 229.9743, found 229.9742.

**(*Z*)-2-Bromo-3-(3-fluoro)phenyl-2-propenal**

72%, *Z : E* = 100 : 4, colorless oil : ^1^H-NMR (CDCl_3_, 400 MHz) δ: 7.22 (dd, *J* = 8 Hz, 6 Hz, 1H), 7.46 (dd, *J* = 14.5, 7 Hz, 1H), 7.69 (d, *J* = 8Hz, 1H), 7.82 (d, *J* = 10 Hz, 1H), 7.88 (s, 1H), 9.36 (s, 1H); ^13^C-NMR (CDCl_3_, 100 MHz) δ: 117.0 (CH, d, *J*^2^_C,F_ = 23 Hz), 118.4 (CH, d, *J*^2^_C,F_ = 21 Hz), 125.4 (C), 127.0 (CH, d, *J*^4^_C,F_ = 2.4 Hz), 130.3 (CH, d, *J*^3^_C,F_ = 8 Hz), 134.8 (CH, d, *J*^3^_C,F_ = 7 Hz), 147.2 (CH), 162.4 (C, d, *J*^1^_C,F_ = 250 Hz), 186.7 (CH); IR (neat) 1693, 3065 cm^-1^; MS (EI) m/z: 228 (M^+^), 230 (M+2), 149 (M-Br); HRMS (EI) *m/z*: calcd for C_9_H_6_OBrF 227.9586, found 227.9586.

**(*E*)-2-(3-fluoro)benzylidene-4-phenylbut-3-ynal**

75%, *Z : E* = 1 : 100, brown oil : ^1^H-NMR (CDCl_3_, 400 MHz) δ: 7.18 (ddd, *J* = 8, 8, 3 Hz, 2H), 7.38-7.48 (m, 4H), 7.50 (s, 1H), 7.60 (d, *J* = 8 Hz, 1H), 7.61 (d, *J* = 6 Hz, 1H), 7.76 (d, *J* = 8 Hz, 1H), 8.06 (d, *J* = 10 Hz, 1H), 9.64 (s, 1H); ^13^C-NMR (CDCl_3_, 100 MHz) δ: 83.0 (C), 101.8 (C), 116.4 (CH, d, *J*^2^_C,F_ = 23 Hz), 118.4 (CH, d, *J*^2^_C,F_ = 22.3 Hz), 122.2 (C), 123.6 (CH), 126.9 (CH), 128.5 (CH), 129.3 (CH), 130.2 (C, *J*^3^_C,F_ = 8.3 Hz), 131.9 (CH), 136.1 (CH, d, *J*^3^_C,F_ = 8.3 Hz), 148.8 (C), 162.6 (C, *J*^1^_C,F_ = 248 Hz), 190.5 (CH); IR (neat) 1693, 2839 cm^-1^; MS (EI) *m/z*: 250 (M^+^); HRMS (EI) *m/z*: calcd for C_17_H_11_OF 250.0794, found 250.0795.

***Z*)-4-((Z)-(3-fluoro)benzylidene)-6-phenylhex-2-en-5-ynoic acid (2o)**

96%; yellow soild (*E:Z* = 5:1): major isomer. ^1^H-NMR (CDCl_3_, 400 MHz) δ: 5.91 (d, *J* = 13 Hz, 1H), 6.61 (d, *J* = 13 Hz, 1H), 6.93 (s, 1H), 7.04 (ddd, *J* = 8, 8, 3 Hz*,* 1H), 7.31-7.37 (m, 4H), 7.49-7.51 (m, 2H), 7.56 (d, *J* = 8 Hz, 1H), 7.88 (d, *J* = 11 Hz, 1H); ^13^C-NMR (CDCl_3_, 150 MHz) δ : 85.9 (C), 98.4 (C), 115.5 (CH, d, *J*^2^_C,F_ = 23 Hz), 116.1 (CH, d, *J*^2^_C,F_ = 22 Hz), 118.0 (C), 120.2 (CH), 122.6 (C), 125.7 (CH, d, *J*^4^_C,F_ = 2.9 Hz), 128.4 (CH), 128.8 (CH), 129.7 (CH, d, *J*^3^_C,F_ = 8.7 Hz), 131.5 (CH,), 137.6 (C, *J*^3^_C,F_ = 8.7 Hz), 140.1 (CH, d, *J*^4^_C,F_ = 2.9 Hz), 140.8 (CH), 162.6 (C, *J*^1^_C,F_ = 244 Hz), 171.8 (C); IR (neat) 1688, 3059 cm^-1^; MS (EI) m/z: 292 (M+), 273 (M-F); HRMS (EI) calcd for C_19_H_13_O_2_H, m/z: 292.0900, found 292.0899.

**Synthesis of 2p**

**(*Z*)-2-Bromo-3-(4-fluoro)phenylprop-2-en-1-ol**

81% (*Z:E* = 100:3), white solid, mp. 33.5-36 °C. ^1^H-NMR (CDCl_3_, 600 MHz) δ:2.28 (-OH, s, 1H), 3.84 (s, 3H), 4.41 (s, 2H), 7.05-7.06 (m, 3H), 7.60 (dd, *J* = 7.9, 5.8 Hz, 2H); ^13^C-NMR (CDCl_3_, 150 MHz) δ: 69.3 (CH_2_), 115.2 (CH_2_, d, *J*_C,F_ = 22 Hz), 125.2 (C), 126.7 (CH_2_), 130.8 (CH, d, *J*^3^_C,F_ = 8.7 Hz), 130.9 (C), 162.3 (C, *J*_C,F_ = 249 Hz); IR (neat) 1604, 1645, 2941, 3400 cm^-1^; MS (EI) m/z: 230 (M^+^), 232 (M+2), HRMS (Fab) calcd for C_9_H_8_OBrF 229.9743, found 229.9743, 231.9742.

**(*Z*)-2-Bromo-3-(4-fluoro)phenyl-2-propenal**

91% (*E:Z* =>99:1), Colorless needles, mp. 60.5-61.5 °C (Et_2_O). ^1^H-NMR (CDCl_3_, 400 MHz) δ: 7.19 (t, *J* = 8.7 Hz, 2H), 7.87 (s, 1H), 8.05 (dd, *J* = 8.2, 5.3 Hz, 2H), 9.34 (s, 1H); ^13^C-NMR (CDCl_3_, 100 MHz) δ: 116.1 (CH, d, *J*_C,F_ = 22 Hz), 123.9 (C), 129.2 (C), 133.2 (CH, d, *J*^2^_C,F_ = 91 Hz), 147.6 (CH), 164.3 (C, *J*^1^_C,F_ = 256 Hz), 186.8 (CH); IR (neat) 1693 cm^-1^; MS (EI) m/z: 228 (M^+^), 230 (M+2); HRMS (EI) calcd for C_9_H_6_OBrF 227.9586, found 227.9585.

**(*E*)-2-(4-Fluoro)benzylidene-4-phenylbut-3-ynal**

62% (*E/Z* = >99/1), Brown oil. ^1^H-NMR (CDCl_3_, 400 MHz) δ: 7.16 (t, *J* = 8.7 Hz, 2H), 7.39-7.40 (m, 3H), 7.49 (s, 1H), 7.58-7.59 (m, 2H), 8.16 (dd, *J*^1^ = 7.2 Hz *J*^2^ = 3.6 Hz, 2H), 9.62 (s, 1H); ^13^C-NMR (CDCl_3_, 100 MHz) δ: 83.0 (C), 100.9 (C), 116.0 (CH, d, *J*_C,F_ = 21 Hz), 122.2 (C), 122.3 (C), 128.5 (CH), 129.2 (CH), 130.5 (C, d, *J*_C,F_ = 2.5 Hz), 131.8 (CH), 132.8 (CH, d, *J*^3^_C,F_ = 9.1 Hz), 149.5 (CH), 164.3 (C, *J*^1^_C,F_ = 256 Hz), 190.7 (CH); IR (neat) 1693, 2204, 3063 cm^-1^; MS (EI) m/z: 250 (M^+^), HRMS (EI) calcd for C_17_H_11_OF, 250.0794, found 250.0795.

**Ethyl (*Z*)-4-((Z)-(4-fluoro)benzylidene)-6-phenylhex-2-en-5-ynoate**

90% *(Z:E* = 10:1), yellow oil. ^1^H-NMR (CDCl_3_, 400 MHz) δ: 1.20 (t, *J* = 6.8 Hz, 3H), 4.13 (q, *J* = 7.1 Hz, 2H), 5.93 (d, *J* = 11.6 Hz, 1H), 6.53 (d, *J* = 11.6 Hz, 1H), 6.92 (s, 1H), 7.08 (t, *J* = 8.2 Hz, 2H), 7.35-7.36 (m, 3H), 7.50 (d, *J* = 3.9 Hz, 2H), 7.95 (dd, *J*^1^ = 8.7 , 5.8 Hz, 2H); ^13^C-NMR (CDCl_3_, 100 MHz) δ : 14.0 (CH_3_), 60.6 (CH_2_), 86.5 (C), 96.9 (C), 115.4 (CH, d, *J*_C,F_ = 22.3 Hz), 116.9 (C), 121.1 (CH), 122.9 (C), 128.4 (CH), 128.7 (CH), 131.3 (CH, d, *J*_C,F_ = 8.3 Hz), 131.5 (CH), 132.0 (C), 138.6 (CH), 139.8 (CH), 162.9 (C, *J*_C,F_ = 252 Hz), 166.8 (C); IR (neat) 1728, 2202, 2982 cm^-1^; MS (EI) *m/z* = 320 (M^+^), 291 (M-Et); HRMS (EI) *m/z:* calcd for C_21_H_17_O_2_F, 320.1213, found 320.1212.

**(*Z*)-4-((Z)-(4-Fluoro)benzylidene)-6-phenylhex-2-en-5-ynoic acid (2p)**

99% (*Z:E* >99:1 after recrystallization), colorless plates, mp. 125.5-128.5 °C (CH_2_Cl_2_/Et_2_O). ^1^H-NMR (CDCl_3_, 400 MHz) δ: 5.86 (d, *J* = 12.6 Hz, 1H), 6.60 (d, *J* = 11.6 Hz, 1H), 6.94 (s, 1H), 7.08 (t, *J* = 8.7 Hz, 2H), 7.33-7.34 (m, 3H), 7.47-7.48 (m, 2H), 7.93-7.97 (m, 2H); ^13^C-NMR (CDCl_3_, 100 MHz) δ : 86.1 (C), 97.5 (C), 115.4 (CH, d, *J*^2^_C,F_ = 21.4 Hz), 116.5 (C), 119.5 (CH), 122.8 (C), 128.4 (CH), 128.7 (CH), 131.4 (CH), 131.5 (CH), 132.0 (C), 140.6 (CH), 141.1 (CH), 163.0 (C, *J*_C,F_ = 252 Hz), 171.6 (C); IR (neat) 1690, 2202, 3032 cm^-1^; MS (EI) *m/z*: 292 (M^+^); HRMS (EI) *m/z:* calcd for C_19_H_13_O_2_F: 292.0900, found 292.0899.

**3. ^1^H and ^13^C NMR spectra**

**2a**

**2b**

**2c**

**2d**

**2e**

**2f**

**2g**

**2h**

**2i (ku-303)**

**2j**

**4.**

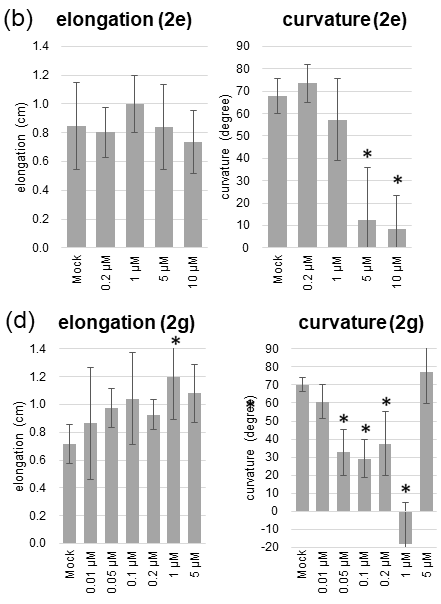

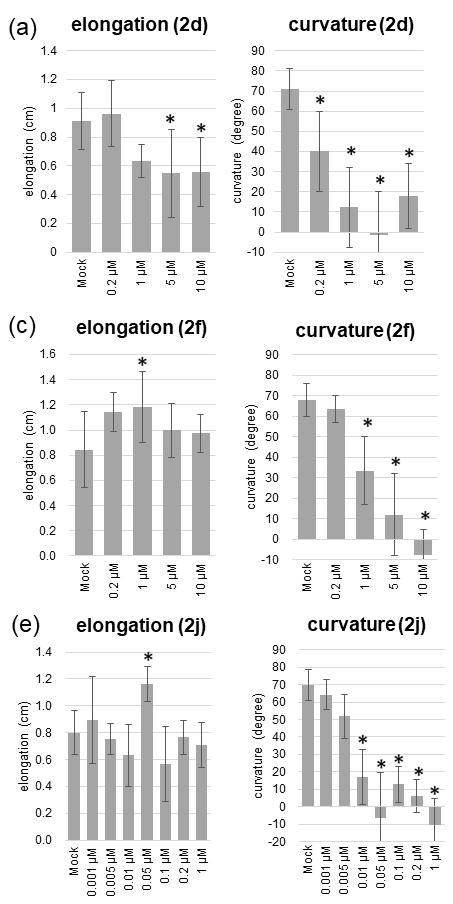


**Figure S1**. Inhibitory activity tests of gravitropic bending and elongation for analogues using Lettuce root. Dose-response relationship study for (a) **2d**, (b) **2e**, (c) **2f**, (d) **2g**, (e) **2j** was shown. Data for gravitropic bending (vertical axis) and elongation (horizontal axis) represent mean ± SD. Asterisk indicates statistically significant differences between treatments and control at p < 0.05 (Turkey-Kramer’s test).

**5.**

**
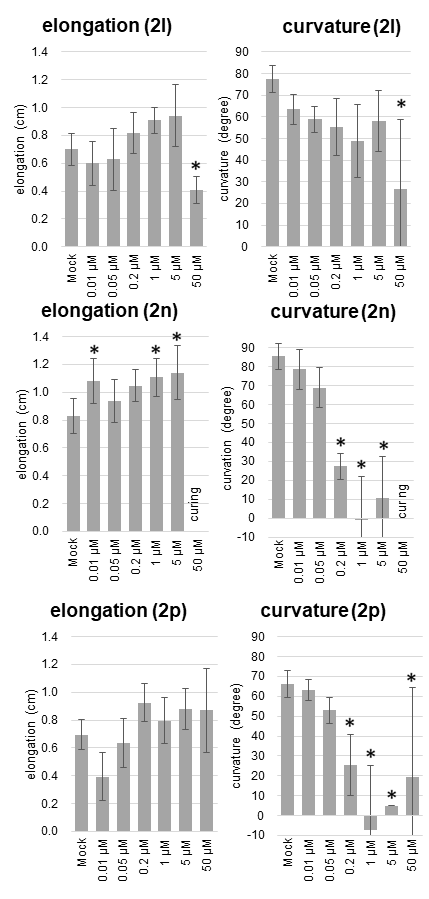

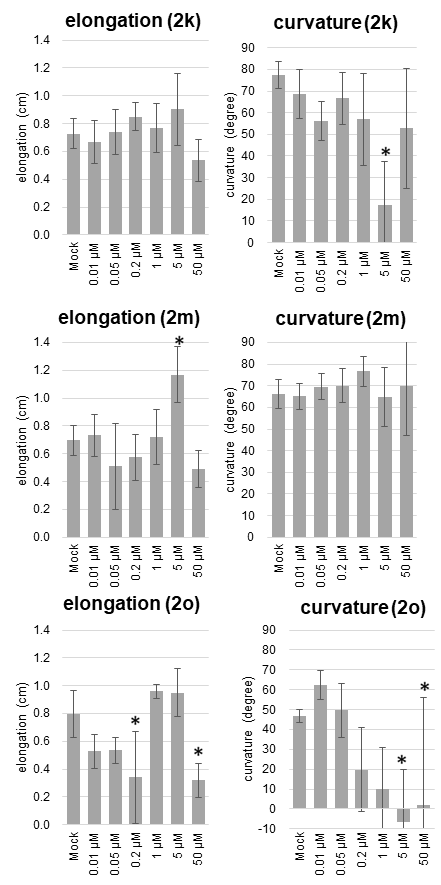
**

**Figure S2.** Inhibitory activity tests of gravitropic bending and elongation for analogues using Lettuce root. Dose-response relationship study for (a) **2k**, (b) **2l**, (c) **2m**, (d) **2n**, (e) **2o**, (f) **2p**, was shown. Data for gravitropic bending (vertical axis) and elongation (horizontal axis) represent mean ± SD. Asterisk indicates statistically significant differences between treatments and control at p < 0.05 (Turkey-Kramer’s test).

**6.**


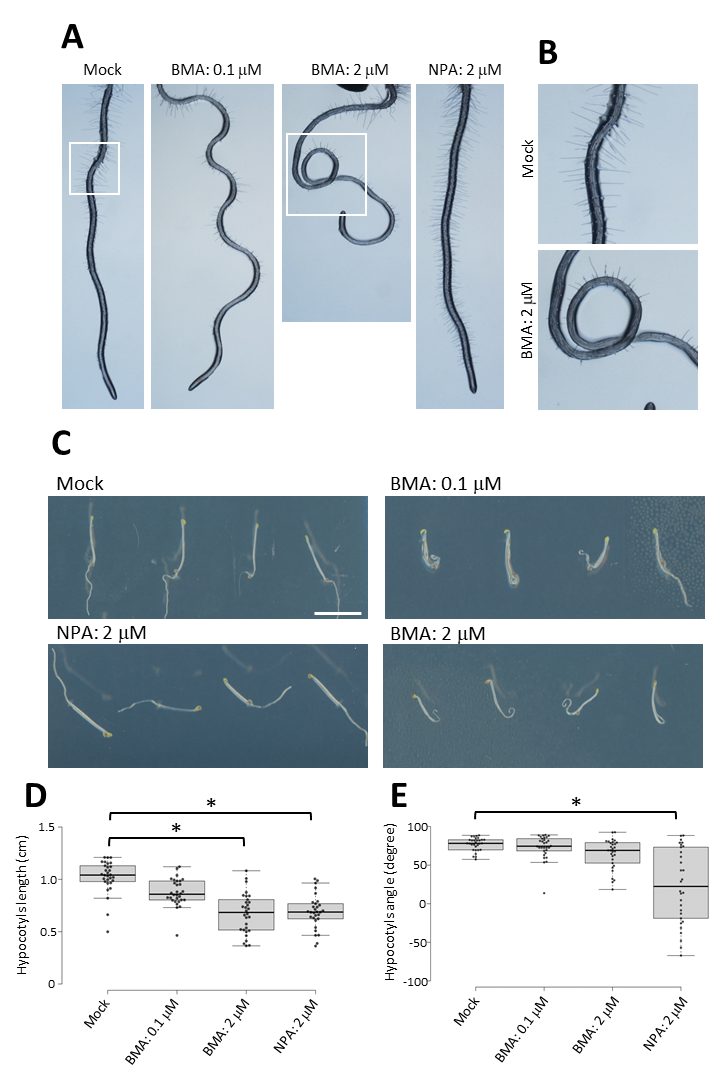


**Figure S3**. (A) and (B) Effect of BMA on root hair formation. (A) *Arabidopsis* Col-0 was grown on a vertical agar plate containing 0.1, 2 μM of BMA and NPA for six days under contentious light conditions. (B) Magnified image of the white squares in (A). (C) Effect of BMA on the elongation and growth angle of the etiolated hypocotyls. *Arabidopsis* Col-0 was grown on a vertical agar plate containing 0.1, 2 μM of BMA and NPA for three days under dark conditions. (D) Length of hypocotyls. Asterisks indicate significant differences between data points (Tukey, *P*<0.05). n=30. (E) Angle of hypocotyls. Upward elongation is shown as positive and downward elongation as negative. Asterisks indicate significant differences between data points (Tukey, *P*<0.05). n=30.

**7.**

**
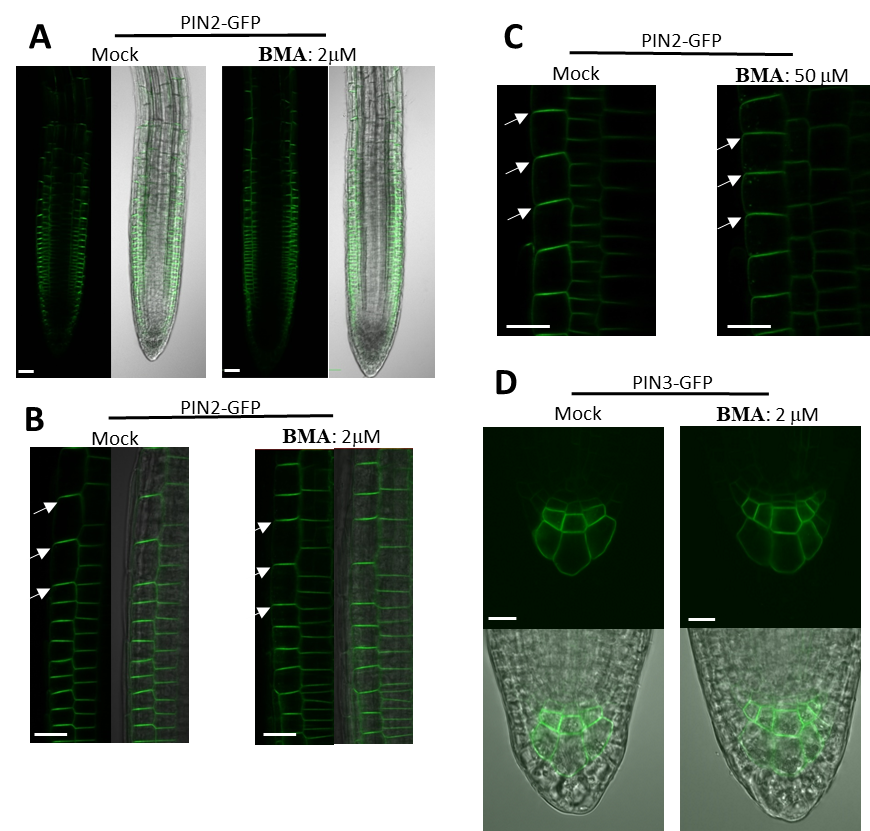
**

**Figure S4**. (A) Evaluation of the effect of BMA on the localization pattern of PIN2-GFP. Arabidopsis PIN2-GFP seedlings were grown on a vertical agar plate containing 2 μM BMA for 6 days under contentious light condition. PIN2-GFP localizes with an upper polarity in epidermal cells from the root meristem zone to the elongation zone. PIN2-GFP localized with an upper polarity in roots of shoots grown in the presence of BMA**,** indicating that BMA does not affect the localization pattern of PIN2-GFP Bar = 30 μm. A magnified image of the epidermis is shown in (B). Arrows indicate PIN2-GFP localized polarized to the upper side of the cells. Bar = 20 μm. Fluorescence signal was measured on upper and outer membranes of epidermal cells. The fluorescence ratio (Upper/outer) was shown. There is no significant difference between indicated data (student *t*-test, *P>*0.05). n=10. (C) Short term treatment with high concentrations of BMA on PIN2-GFP. PIN2-GFP plants were grown for 6 days under no inhibitor conditions. The seedlings were then treated with 50 μM BMA in MS liquid medium for 90 min. Bar = 20 μm. There is no significant difference in fluorescence ratio (upper/outer) between indicated data (student *t*-test, *P>*0.05). n=10. (D) Short term treatment with BMA on PIN3-GFP. PIN3-GFP plants were grown for 6 days under no inhibitor conditions. The seedlings were then treated with 50 μM BMA in MS liquid medium for 90 min. Bar = 20 μm. The intensity of PIN3-GFP in columella cells were compared between mock and BMA treated roots. There is no significant difference between indicated data (student *t*-test, *P>*0.05). n=10.

**8. Raw data of gravitropism bending inhibition tests**

**[Lettuce]**

**7.1 ku-76**

| elongation | Control | ku-76 | | |
| --- | --- | --- | --- | --- |
| length cm |  | 1 μM | 5 μM | 10 μM |
| 1 | 0.86 | 1.4 | 1.11 | 1.26 |
| 2 | 1.03 | 0.84 | 1.34 | 1.25 |
| 3 | 1.28 | 1.06 | 0.8 | 1.08 |
| 4 | 0.84 | 1.33 | 0.95 | 0.66 |
| 5 | 0.74 | 1.02 | 1.02 | 0.78 |
| 6 | 0.71 | 1.25 | 0.8 | 0.8 |
| 7 | 0.93 | 0.87 | 1.05 | 1.23 |
| average | 0.91 | 1.11 | 1.01 | 1.01 |
| SD | 0.20 | 0.22 | 0.19 | 0.26 |

| curvature | Control | ku-76 | | |
| --- | --- | --- | --- | --- |
| θ° |  | 1 μM | 5 μM | 10 μM |
| 1 | 72 | 73 | 26 | 16 |
| 2 | 72 | 33 | 27 | 16 |
| 3 | 77 | 50 | 31 | 18 |
| 4 | 80 | 55 | 0 | 20 |
| 5 | 77 | 50 | 36 | 31 |
| 6 | 49 | 63 | 24 | 12 |
| 7 | 70 | 72 | 31 | 25 |
| average | 71.00 | 56.57 | 25.00 | 19.71 |
| SD | 10.33 | 14.11 | 11.72 | 6.40 |

**7.2 NPA**

| elongation | Control | | NPA | | | |
| --- | --- | --- | --- | --- | --- | --- |
| length cm |  |  | 0.05 μM | 0.2 μM | 1 μM | 5 μM |
| 1 | 0.89 | 0.93 | 0.73 | 0.74 | 0.35 | 0.31 |
| 2 | 0.66 | 0.97 | 0.27 | 0.15 | 0.43 | 0.22 |
| 3 | 1.15 | 1.12 | 0.58 | 0.34 | 0.57 | 0.16 |
| 4 | 1.09 | 0.82 | 0.66 | 0.55 | 0.62 | 0.16 |
| 5 | 0.64 | 0.71 | 0.63 | 0.33 | 0.13 | 0.31 |
| 6 | 0.13 | 0.94 | 0.54 | 0.38 | 0.46 | 0.32 |
| 7 | 1.35 | 0.81 | 0.64 | 0.21 | 0.27 | 0.27 |
| average | 0.9 | | 0.6 | 0.4 | 0.4 | 0.2 |
| SD | 0.3 | | 0.2 | 0.2 | 0.2 | 0.1 |

| curvature | Control | | NPA | | | |
| --- | --- | --- | --- | --- | --- | --- |
| θ° |  |  | 0.05 μM | 0.2 μM | 1 μM | 5 μM |
| 1 | 72 | 74 | 38 | 16 | -18 | 1 |
| 2 | 68 | 84 | 39 | 16 | 9 | -4 |
| 3 | 55 | 71 | 33 | -14 | 12 | -37 |
| 4 | 78 | 80 | 25 | 9 | -6 | -12 |
| 5 | 74 | 79 | 56 | 13 | 0 | -7 |
| 6 | 74 | 73 | 23 | -19 | 1 | 10 |
| 7 | 80 | 78 | 68 | 21 | -20 | -8 |
| average | 74.3 | | 40.3 | 6.0 | -3.1 | -8.1 |
| SD | 7.0 | | 16.4 | 15.9 | 12.4 | 14.6 |

**7.3 2a**

| elongation | Control | | **2a** | | | | | | | |
| --- | --- | --- | --- | --- | --- | --- | --- | --- | --- | --- |
| length cm |  |  | 0.01 μM | 0.05 μM | 0.1 μM | 0.2 μM | 1 μM | 5 μM | 10 μM | 50 μM |
| 1 | 0.81 | 0.86 | 0.79 | 0.59 | 1.03 | 0.98 | 1.39 | 0.84 | 1.03 | 1.02 |
| 2 | 0.55 | 1.03 | 0.73 | 0.71 | 0.97 | 1.01 | 0.88 | 0.89 | 1.12 | 1.07 |
| 3 | 0.77 | 1.28 | 0.70 | 0.59 | 0.87 | 0.88 | 0.96 | 0.92 | 0.83 | 0.76 |
| 4 | 0.59 | 0.84 | 0.62 | 0.88 | 1.20 | 0.93 | 0.93 | 0.82 | 0.98 | 1.04 |
| 5 | 0.76 | 0.74 | 0.55 | 0.56 | 0.65 | 1.01 | 1.13 | 0.99 | 0.89 | 1.01 |
| 6 | 1.01 | 0.71 | 0.97 | 0.65 | 0.82 | 0.97 | 1.10 | 1.44 | 0.44 |  |
| 7 | 0.76 | 0.93 | 0.72 | 1.06 | 0.70 | 0.96 | 1.07 | 0.77 | 0.66 |  |
| average | 0.83 | | 0.73 | 0.72 | 0.89 | 0.96 | 1.07 | 0.95 | 0.85 | 0.98 |
| SD | 0.19 | | 0.13 | 0.19 | 0.19 | 0.04 | 0.17 | 0.23 | 0.23 | 0.13 |

| curvature | Control | | **2a** | | | | | | | |
| --- | --- | --- | --- | --- | --- | --- | --- | --- | --- | --- |
| θ° |  |  | 0.01 μM | 0.05 μM | 0.1 μM | 0.2 μM | 1 μM | 5 μM | 10 μM | 50 μM |
| 1 | 76 | 72 | 60 | 34 | 43 | 12 | 16 | -1 | 16 | 16 |
| 2 | 68 | 72 | 67 | 65 | 38 | 34 | -15 | 12 | 16 | -16 |
| 3 | 66 | 77 | 56 | 61 | 52 | 20 | 10 | 24 | 17 | -3 |
| 4 | 71 | 80 | 69 | 74 | 62 | 14 | 4 | 16 | 48 | 16 |
| 5 | 73 | 77 | 58 | 53 | 38 | 8 | -15 | 5 | 17 | 47 |
| 6 | 66 | 49 | 68 | 53 | 53 | 28 | 14 | -31 | 18 |  |
| 7 | 70 | 70 | 63 | 56 | 29 | 22 | -15 | 3 | 17 |  |
| average | 70.5 | | 63.0 | 56.6 | 45.0 | 19.7 | -0.1 | 4.0 | 21.3 | 12.0 |
| SD | 7.5 | | 5.2 | 12.4 | 11.3 | 9.2 | 14.4 | 17.6 | 11.8 | 23.8 |

**7.4 2b**

| elongation | Control | | **2b** | | | | | | |  |
| --- | --- | --- | --- | --- | --- | --- | --- | --- | --- | --- |
| L cm |  |  | 0.01 μM | 0.05 μM | 0.2 μM | 1 μM | 5 μM | 10 μM | 50 μM | |
| 1 | 0.81 | 0.86 | 0.87 | 0.59 | 0.75 | 0.27 | 1.06 | 0.11 | 0.34 | |
| 2 | 0.55 | 1.03 | 1.24 | 0.24 | 0.86 | 0.59 | 0.86 | 0.62 | 0.24 | |
| 3 | 0.77 | 1.28 | 1.53 | 0.35 | 0.74 | 0.96 | 0.98 | 0.73 | 0.07 | |
| 4 | 0.59 | 0.84 | 0.98 | 0.62 | 0.85 | 0.54 | 0.99 | 0.66 | 0.18 | |
| 5 | 0.76 | 0.74 | 0.92 | 0.90 | 0.64 | 0.75 | 0.80 | 0.64 | 0.27 | |
| 6 | 1.01 | 0.71 | 0.60 | 0.67 | 0.73 | 0.82 | 0.71 | 0.57 | 0.31 | |
| 7 | 0.76 | 0.93 | 0.85 | 0.11 | 0.97 | 0.85 | 0.89 | 0.57 | 0.40 | |
| average | 0.83 | | 1.00 | 0.50 | 0.79 | 0.68 | 0.90 | 0.56 | 0.26 | |
| SD | 0.19 | | 0.30 | 0.27 | 0.11 | 0.23 | 0.12 | 0.21 | 0.11 | |

| curvature | Control | | **2b** | | | | | | |  |
| --- | --- | --- | --- | --- | --- | --- | --- | --- | --- | --- |
| θ° |  |  | 0.01 μM | 0.05 μM | 0.2 μM | 1 μM | 5 μM | 10 μM | 50 μM | |
| 1 | 76 | 72 | 61 | 37 | 3 | 10 | -25 | -26 | 15 | |
| 2 | 68 | 72 | 57 | 14 | -13 | 6 | 17 | -15 | 0 | |
| 3 | 66 | 77 | 35 | 32 | 31 | 28 | -6 | 9 | 51 | |
| 4 | 71 | 80 | 52 | 20 | 27 | 3 | -13 | 66 | 26 | |
| 5 | 73 | 77 | 48 | 32 | -12 | 4 | 20 | -27 | -17 | |
| 6 | 66 | 49 | 55 | 31 | 14 | -13 | -13 | -36 | -4 | |
| 7 | 70 | 70 | 61 | 21 | 6 | 3 | -9 | -77 | 16 | |
| average | 70.5 | | 52.7 | 26.7 | 8.0 | 5.9 | -4.1 | -15.1 | 12.4 | |
| SD | 7.5 | | 9.1 | 8.4 | 17.3 | 12.1 | 16.6 | 44.1 | 22.3 | |

**7.5 2c**

| elongation | Control | | **2c** | | | | | | | |
| --- | --- | --- | --- | --- | --- | --- | --- | --- | --- | --- |
| L cm |  |  | 0.01 μM | 0.05 μM | 0.1 μM | 0.2 μM | 1 μM | 5 μM | 10 μM | 50 μM |
| 1 | 0.81 | 0.86 | 1.31 | 0.46 | 0.50 | 0.97 | 0.80 | -0.04 | 0.46 | 0.27 |
| 2 | 0.55 | 1.03 | 0.82 | 0.84 | 0.78 | 1.04 | 0.90 | 0.08 | 0.63 | 0.15 |
| 3 | 0.77 | 1.28 | 0.89 | 0.73 | 0.34 | 0.96 | 1.22 | 0.01 | 0.49 | 0.33 |
| 4 | 0.59 | 0.84 | 0.74 | 0.74 | 0.89 | 0.80 | 1.14 | 0.18 | 0.60 | 0.27 |
| 5 | 0.76 | 0.74 | 1.03 | 0.99 | 0.63 | 0.99 | 1.20 | 0.11 | 0.64 | 0.00 |
| 6 | 1.01 | 0.71 | 0.98 | 0.71 | 0.79 | 0.70 | 1.07 | 0.24 | 0.46 | 0.15 |
| 7 | 0.76 | 0.93 | 1.10 | 0.54 | 0.40 | 0.88 | 0.78 | 0.20 | 0.50 |  |
| average | 0.83 | | 0.98 | 0.71 | 0.62 | 0.90 | 1.02 | 0.11 | 0.54 | 0.20 |
| SD | 0.19 | | 0.19 | 0.18 | 0.21 | 0.12 | 0.19 | 0.10 | 0.08 | 0.12 |

| curvature | Control | | **2c** | | | | | | | |
| --- | --- | --- | --- | --- | --- | --- | --- | --- | --- | --- |
| θ° |  |  | 0.01 μM | 0.05 μM | 0.1 μM | 0.2 μM | 1 μM | 5 μM | 10 μM | 50 μM |
| 1 | 76 | 72 | 55 | 10 | -1 | 21 | -6 | 19 | 12 | 42 |
| 2 | 68 | 72 | 48 | 24 | 22 | -18 | 19 | -36 | 10 | 7 |
| 3 | 66 | 77 | 53 | 37 | -8 | 10 | -17 | -20 | -16 | 35 |
| 4 | 71 | 80 | 59 | 32 | 5 | -17 | 14 | -22 | 19 | 8 |
| 5 | 73 | 77 | 49 | 47 | 41 | 6 | 3 | 3 | 10 | 0 |
| 6 | 66 | 49 | 50 | 37 | 22 | 17 | 19 | 7 | 19 | 0 |
| 7 | 70 | 70 | 69 | 36 | -14 | 7 | 11 | 16 | 2 |  |
| average | 70.5 | | 54.7 | 31.9 | 9.6 | 3.7 | 6.1 | -4.7 | 8.0 | 15.3 |
| SD | 7.5 | | 7.4 | 11.8 | 19.6 | 15.4 | 13.6 | 21.2 | 12.1 | 18.4 |

**7.6 2d**

| elongation | Control | **2d** | | | |  |
| --- | --- | --- | --- | --- | --- | --- |
| cm |  | 0.2 μM | 1 μM | 5 μM | 10 μM | |
| 1 | 0.86 | 1.02 | 0.68 | 0.67 | 0.66 | |
| 2 | 1.03 | 0.75 | 0.49 | 0.92 | 0.82 | |
| 3 | 1.28 | 1.14 | 0.72 | 0.54 | 0.84 | |
| 4 | 0.84 | 1.23 | 0.75 | 0.16 | 0.45 | |
| 5 | 0.74 | 1.16 | 0.66 | 0.62 | 0.56 | |
| 6 | 0.71 | 0.77 | 0.45 | 0.12 | 0.34 | |
| 7 | 0.93 | 0.67 | 0.68 | 0.80 | 0.21 | |
| average | 0.91 | 0.96 | 0.63 | 0.55 | 0.56 | |
| SD | 0.20 | 0.23 | 0.11 | 0.31 | 0.24 | |

| curvature | Control | **2d** | | | |  |
| --- | --- | --- | --- | --- | --- | --- |
| θ° |  | 0.2 μM | 1 μM | 5 μM | 10 μM | |
| 1 | 72 | 65 | -11 | -4 | -6 | |
| 2 | 72 | 59 | 20 | -28 | 13 | |
| 3 | 77 | 22 | 17 | 15 | 2 | |
| 4 | 80 | 17 | -20 | -29 | 29 | |
| 5 | 77 | 23 | 31 | 28 | 18 | |
| 6 | 49 | 54 | 20 | -3 | 38 | |
| 7 | 70 | 40 | 29 | 11 | 31 | |
| average | 71.0 | 40.0 | 12.3 | -1.4 | 17.9 | |
| SD | 10.3 | 19.7 | 19.8 | 21.5 | 16.1 | |

**7.7 2e**

| elongation | Control | | **2e** | | | |
| --- | --- | --- | --- | --- | --- | --- |
| cm |  |  | 0.2 μM | 1 μM | 5 μM | 10 μM |
| 1 | 0.89 | 1.02 | 0.85 | 1.02 | 1.17 | 1.12 |
| 2 | 0.66 | 0.78 | 0.92 | 1.13 | 0.71 | 0.86 |
| 3 | 1.15 | 0.79 | 0.85 | 0.96 | 0.39 | 0.56 |
| 4 | 1.09 | 1.02 | 1.01 | 1.07 | 0.89 | 0.56 |
| 5 | 0.64 | 0.79 | 0.86 | 0.63 | 0.56 | 0.63 |
| 6 | 0.13 | 0.68 | 0.52 | 0.95 | 1.16 | 0.57 |
| 7 | 1.35 |  | 0.62 | 1.26 | 0.99 | 0.87 |
| average | 0.84 | | 0.80 | 1.00 | 0.84 | 0.74 |
| SD | 0.30 | | 0.17 | 0.20 | 0.30 | 0.22 |

| curvature | Control | | **2e** | | | |
| --- | --- | --- | --- | --- | --- | --- |
| θ° |  |  | 0.2 μM | 1 μM | 5 μM | 10 μM |
| 1 | 72 | 59 | 79 | 71 | 27 | 16 |
| 2 | 68 | 68 | 68 | 53 | 6 | -4 |
| 3 | 55 | 62 | 59 | 80 | -16 | -4 |
| 4 | 78 | 56 | 81 | 38 | -16 | 14 |
| 5 | 74 | 69 | 74 | 66 | 17 | -2 |
| 6 | 74 | 67 | 70 | 29 | 19 | 3 |
| 7 | 80 |  | 83 | 64 | 50 | 37 |
| average | 67.8 | | 73.4 | 57.3 | 12.4 | 8.6 |
| SD | 8.0 | | 8.5 | 18.3 | 23.6 | 15.0 |

**7.8 2f**

| elongation | Control | | **2f** | | | |
| --- | --- | --- | --- | --- | --- | --- |
| cm |  |  | 0.2 μM | 1 μM | 5 μM | 10 μM |
| 1 | 0.89 | 1.02 | 1.08 | 0.97 | 1.14 | 0.94 |
| 2 | 0.66 | 0.78 | 1.02 | 0.90 | 0.92 | 1.25 |
| 3 | 1.15 | 0.79 | 1.37 | 1.01 | 0.86 | 0.89 |
| 4 | 1.09 | 1.02 | 1.23 | 1.52 | 1.43 | 0.87 |
| 5 | 0.64 | 0.79 | 1.00 | 1.34 | 0.94 | 0.80 |
| 6 | 0.13 | 0.68 | 1.30 | 1.57 | 0.81 | 1.08 |
| 7 | 1.35 |  | 1.01 | 1.00 | 0.91 | 0.97 |
| average | 0.84 | | 1.14 | 1.18 | 1.00 | 0.97 |
| SD | 0.30 | | 0.16 | 0.28 | 0.22 | 0.15 |

| curvature | Control | | **2e** | | | |  |
| --- | --- | --- | --- | --- | --- | --- | --- |
| θ° |  |  | 0.2 μM | 1 μM | 5 μM | 10 μM | |
| 1 | 72 | 59 | 79 | 71 | 27 | 16 | |
| 2 | 68 | 68 | 68 | 53 | 6 | -4 | |
| 3 | 55 | 62 | 59 | 80 | -16 | -4 | |
| 4 | 78 | 56 | 81 | 38 | -16 | 14 | |
| 5 | 74 | 69 | 74 | 66 | 17 | -2 | |
| 6 | 74 | 67 | 70 | 29 | 19 | 3 | |
| 7 | 80 |  | 83 | 64 | 50 | 37 | |
| average | 67.8 | | 73.4 | 57.3 | 12.4 | 8.6 | |
| SD | 8.0 | | 8.5 | 18.3 | 23.6 | 15.0 | |

**7.9 2g**

| elongation | Control | | **2g** | | | | | |
| --- | --- | --- | --- | --- | --- | --- | --- | --- |
| cm |  |  | 0.01 μM | 0.05 μM | 0.1 μM | 0.2 μM | 1 μM | 5 μM |
| 1 | 0.81 | 0.71 | 1.52 | 0.93 | 0.85 | 1.02 | 1.01 | 1.00 |
| 2 | 0.55 | 0.62 | 0.17 | 0.89 | 1.57 | 1.12 | 0.84 | 1.47 |
| 3 | 0.77 | 0.76 | 0.89 | 1.07 | 1.14 | 0.92 | 1.11 | 1.10 |
| 4 | 0.59 | 0.49 | 0.76 | 0.73 | 1.12 | 0.85 | 1.00 | 0.91 |
| 5 | 0.76 | 0.64 | 0.78 | 1.01 | 0.93 | 0.83 | 1.34 | 0.91 |
| 6 | 1.01 | 0.67 | 1.04 | 1.17 | 0.50 | 0.89 | 1.76 | 1.10 |
| 7 | 0.76 | 0.91 | 0.90 | 1.00 | 1.16 | 0.85 | 1.31 |  |
| average | 0.72 | | 0.86 | 0.97 | 1.04 | 0.93 | 1.19 | 1.08 |
| SD | 0.14 | | 0.40 | 0.14 | 0.33 | 0.11 | 0.30 | 0.21 |

| curvature | Control | | **2g** | | | | | |
| --- | --- | --- | --- | --- | --- | --- | --- | --- |
| θ° |  |  | 0.01 μM | 0.05 μM | 0.1 μM | 0.2 μM | 1 μM | 5 μM |
| 1 | 76 | 74 | 57 | 51 | 27 | 23 | -6 | 45 |
| 2 | 68 | 63 | 56 | 34 | 32 | 25 | -22 | 89 |
| 3 | 66 | 69 | 78 | 29 | 34 | 36 | 13 | 73 |
| 4 | 71 | 66 | 50 | 20 | 17 | 40 | -38 | 75 |
| 5 | 73 | 71 | 54 | 29 | 42 | 68 | -40 | 88 |
| 6 | 66 | 75 | 66 | 48 | 38 | 52 | 7 | 92 |
| 7 | 70 | 72 | 64 | 18 | 14 | 18 | -39 |  |
| average | 70 | | 61 | 33 | 29 | 37 | -18 | 77 |
| SD | 4 | | 9 | 13 | 10 | 18 | 23 | 18 |

**7.10 2h**

| elongation | Control | | | | **2h** | | | | | | | |
| --- | --- | --- | --- | --- | --- | --- | --- | --- | --- | --- | --- | --- |
| cm |  |  |  |  | 0.001 μM | 0.005 μM | 0.01 μM | 0.05 μM | 0.1 μM | 0.2 μM | 1 μM | 5 μM |
| 1 | 0.81 | 0.71 | 0.89 | 1.02 | 0.97 | 0.64 | 0.77 | 0.90 | 0.78 | 1.37 | 1.50 | 0.92 |
| 2 | 0.55 | 0.62 | 0.66 | 0.78 | 0.75 | 0.59 | 0.75 | 0.89 | 1.12 | 0.99 | 1.18 | 0.62 |
| 3 | 0.77 | 0.76 | 1.15 | 0.79 | 0.51 | 0.98 | 1.07 | 1.02 | 0.80 | 0.73 | 1.00 | 0.35 |
| 4 | 0.59 | 0.49 | 1.09 | 1.02 | 0.71 | 0.66 | 0.79 | 1.20 | 0.66 | 0.73 | 0.89 | 0.79 |
| 5 | 0.76 | 0.64 | 0.64 | 0.79 | 0.64 | 0.94 | 0.87 | 0.84 | 0.80 | 1.02 | 0.83 | 1.40 |
| 6 | 1.01 | 0.67 | 0.13 | 0.68 | 0.99 | 0.80 | 0.89 | 1.06 | 0.73 | 1.00 | 1.17 | 0.70 |
| 7 | 0.76 | 0.91 | 1.35 |  | 1.14 | 0.84 | 0.47 | 0.92 | 0.67 | 1.01 | 1.08 |  |
| average | 0.78 | | | | 0.82 | 0.78 | 0.80 | 0.98 | 0.80 | 0.98 | 1.09 | 0.80 |
| SD | 0.24 | | | | 0.22 | 0.15 | 0.18 | 0.12 | 0.16 | 0.22 | 0.22 | 0.35 |

| curvature | Control | | | | 2h | | | | | | | |
| --- | --- | --- | --- | --- | --- | --- | --- | --- | --- | --- | --- | --- |
| θ° |  |  |  |  | 0.001 μM | 0.005 μM | 0.01 μM | 0.05 μM | 0.1 μM | 0.2 μM | 1 μM | 5 μM |
| 1 | 76 | 74 | 72 | 59 | 40 | 41 | 6 | 4 | -37 | -29 | -41 | -9 |
| 2 | 68 | 63 | 68 | 68 | 55 | 45 | -5 | -18 | 20 | -14 | -57 | -34 |
| 3 | 66 | 69 | 55 | 62 | 47 | 75 | -4 | -5 | -11 | -16 | -73 | 16 |
| 4 | 71 | 66 | 78 | 56 | 52 | 50 | -2 | -13 | 36 | -23 | 18 | -30 |
| 5 | 73 | 71 | 74 | 69 | 57 | 65 | 13 | -4 | -7 | -27 | -11 | -1 |
| 6 | 66 | 75 | 74 | 67 | 44 | 55 | -10 | -14 | -7 | 29 | -52 | -6 |
| 7 | 70 | 72 | 80 |  | 70 | 67 | 8 | 16 | 10 | 16 | -23 |  |
| average | 69 | | | | 52 | 57 | 1 | -5 | 1 | -9 | -34 | -11 |
| SD | 6 | | | | 10 | 13 | 8 | 12 | 24 | 23 | 31 | 19 |

**7.11 BMA**

| elongation | Control | | BMA | | | | | |
| --- | --- | --- | --- | --- | --- | --- | --- | --- |
| cm |  |  | 0.001 μM | 0.005 μM | 0.01 μM | 0.05 μM | 0.1 μM | 0.2 μM |
| 1 | 0.61 | 1.01 | 0.76 | 0.83 | 0.79 | 0.68 | 0.85 | 1.05 |
| 2 | 0.85 | 0.71 | 0.86 | 0.88 | 0.80 | 0.70 | 0.70 | 1.07 |
| 3 | 0.97 | 0.79 | 0.64 | 0.55 | 0.87 | 0.76 | 0.68 | 1.01 |
| 4 | 1.00 | 0.58 | 0.66 | 0.56 | 0.87 | 0.81 | 0.79 | 1.17 |
| 5 | 1.14 | 0.59 | 0.70 | 0.54 | 0.82 | 0.73 | 0.96 | 0.99 |
| 6 | 0.79 | 0.76 | 0.95 | 0.69 | 0.87 | 0.67 | 0.80 | 0.92 |
| 7 |  | 0.84 | 0.91 |  | 0.82 | 0.76 | 0.84 | 1.16 |
| average | 0.80 | | 0.78 | 0.67 | 0.84 | 0.73 | 0.80 | 1.05 |
| SD | 0.17 | | 0.13 | 0.15 | 0.04 | 0.05 | 0.09 | 0.09 |

| curvature | Control | | BMA | | | | | |
| --- | --- | --- | --- | --- | --- | --- | --- | --- |
| θ° |  |  | 0.001 μM | 0.005 μM | 0.01 μM | 0.05 μM | 0.1 μM | 0.2 μM |
| 1 | 68 | 79 | 43 | 48 | 26 | 4 | 1 | 20 |
| 2 | 74 | 65 | 63 | 68 | 3 | -20 | 11 | 41 |
| 3 | 77 | 66 | 47 | 12 | -4 | 7 | 5 | -15 |
| 4 | 74 | 61 | 64 | 57 | -2 | 22 | -2 | -35 |
| 5 | 71 | 50 | 57 | 65 | 13 | 15 | 8 | 4 |
| 6 | 76 | 64 | 63 | 47 | 10 | 15 | 10 | 27 |
| 7 |  | 82 | 61 |  | -6 | -9 | -6 | 24 |
| average | 70 | | 57 | 50 | 6 | 5 | 4 | 9 |
| SD | 9 | | 8 | 20 | 11 | 15 | 6 | 27 |

**7.12 2j**

| elongation | Control | | **2j** | | | | | | |
| --- | --- | --- | --- | --- | --- | --- | --- | --- | --- |
| cm |  |  | 0.001 μM | 0.005 μM | 0.01 μM | 0.05 μM | 0.1 μM | 0.2 μM | 1 μM |
| 1 | 1.01 | 0.61 | 0.56 | 0.78 | 0.76 | 0.97 | 0.58 | 0.80 | 1.00 |
| 2 | 0.71 | 0.85 | 1.12 | 0.75 | 0.83 | 1.20 | 0.57 | 0.83 | 0.49 |
| 3 | 0.79 | 0.97 | 0.72 | 0.90 | 0.23 | 1.07 | 0.91 | 0.89 | 0.58 |
| 4 | 0.58 | 1 | 0.64 | 0.82 | 0.74 | 1.36 | 0.62 | 0.74 | 0.63 |
| 5 | 0.59 | 1.14 | 1.41 | 0.80 | 0.82 | 1.13 | 0.72 | 0.89 | 0.69 |
| 6 | 0.76 | 0.79 | 0.67 | 0.54 | 0.38 | 1.12 | 0.57 | 0.55 | 0.75 |
| 7 | 0.84 |  | 1.14 | 0.68 | 0.65 | 1.28 | 0 | 0.67 | 0.82 |
| average | 0.80 | | 0.89 | 0.75 | 0.63 | 1.16 | 0.57 | 0.77 | 0.71 |
| SD | 0.17 | | 0.32 | 0.11 | 0.23 | 0.13 | 0.28 | 0.12 | 0.17 |

| curvature | Control | | **2j** | | | | | | |
| --- | --- | --- | --- | --- | --- | --- | --- | --- | --- |
| θ° |  |  | 0.001 μM | 0.005 μM | 0.01 μM | 0.05 μM | 0.1 μM | 0.2 μM | 1 μM |
| 1 | 79 | 68 | 61 | 48 | 33 | -24 | 22 | 19 | -27 |
| 2 | 65 | 74 | 71 | 48 | 17 | 11 | 10 | 0 | -22 |
| 3 | 66 | 77 | 66 | 69 | 9 | -32 | 26 | 6 | 8 |
| 4 | 61 | 74 | 55 | 44 | 10 | 8 | 22 | -9 | -10 |
| 5 | 50 | 71 | 66 | 59 | -7 | 6 | 3 | 5 | -23 |
| 6 | 64 | 76 | 53 | 32 | 18 | -43 | 8 | 16 | 9 |
| 7 | 82 |  | 78 | 63 | 40 | 28 | 0 | 7 | -6 |
| average | 70 | | 64 | 52 | 17 | -7 | 13 | 6 | -10 |
| SD | 9 | | 9 | 13 | 16 | 26 | 10 | 9 | 15 |

**7.13 2k**

| elongation | Control | **2k** | | | | | |
| --- | --- | --- | --- | --- | --- | --- | --- |
| cm |  | 0.01 μM | 0.05 μM | 0.2 μM | 1 μM | 5 μM | 50 μM |
| 1 | 0.81 | 0.81 | 0.75 | 0.91 | 0.82 | 0.82 | 0.70 |
| 2 | 0.74 | 0.81 | 0.91 | 0.67 | 0.67 | 0.92 | 0.55 |
| 3 | 0.85 | 0.67 | 0.72 | 0.92 | 0.51 | 0.54 | 0.51 |
| 4 | 0.61 | 0.44 | 0.69 | 0.98 | 0.86 | 1.15 | 0.30 |
| 5 | 0.62 | 0.61 | 0.88 | 0.88 | 0.96 | 1.24 | 0.45 |
| 6 | - | - | 0.46 | 0.80 | - | 0.76 | 0.70 |
| 7 | - | - | - | 0.80 | - | - | - |
| average | 0.73 | 0.67 | 0.74 | 0.85 | 0.77 | 0.90 | 0.54 |
| SD | 0.11 | 0.16 | 0.16 | 0.10 | 0.18 | 0.26 | 0.15 |

| curvature | Control | **2k** | | | | | |
| --- | --- | --- | --- | --- | --- | --- | --- |
| θ° |  | 0.01 μM | 0.05 μM | 0.2 μM | 1 μM | 5 μM | 50 μM |
| 1 | 73 | 57 | 58 | 52 | 78 | 17 | 68 |
| 2 | 88 | 81 | 69 | 88 | 49 | 10 | 26 |
| 3 | 73 | 67 | 61 | 76 | 83 | 14 | 41 |
| 4 | 81 | 84 | 56 | 58 | 38 | 42 | 42 |
| 5 | 77 | 62 | 45 | 68 | 63 | 5 | 100 |
| 6 | 72 | 61 | 61 | 63 | 31 | -10 | 23 |
| 7 | - | - | 44 | 62 | - | 45 | 69 |
| average | 77 | 69 | 56 | 67 | 57 | 18 | 53 |
| SD | 6 | 11 | 9 | 12 | 21 | 20 | 28 |

**7.14 2l**

| elongation | Control | **2l** | | | | | |
| --- | --- | --- | --- | --- | --- | --- | --- |
| cm |  | 0.01 μM | 0.05 μM | 0.2 μM | 1 μM | 5 μM | 50 μM |
| 1 | 0.57 | 0.55 | 0.25 | 0.88 | 0.91 | 0.95 | 0.32 |
| 2 | 0.81 | 0.40 | 0.58 | 0.62 | 1.00 | 0.88 | 0.47 |
| 3 | 0.74 | 0.40 | 0.56 | 0.66 | 1.01 | 0.68 | 0.28 |
| 4 | 0.85 | 0.80 | 0.90 | 0.85 | 0.85 | 1.31 | 0.42 |
| 5 | 0.61 | 0.68 | 0.74 | 0.75 | 0.90 | 0.79 | 0.34 |
| 6 | 0.62 | 0.64 | 0.74 | 1.01 | 0.76 | 1.04 | 0.46 |
| 7 | - | 0.73 | - | 0.95 | - | - | 0.56 |
| average | 0.70 | 0.60 | 0.63 | 0.82 | 0.91 | 0.94 | 0.41 |
| SD | 0.12 | 0.16 | 0.22 | 0.14 | 0.09 | 0.22 | 0.10 |

| curvature | Control | **2l** | | | | | |
| --- | --- | --- | --- | --- | --- | --- | --- |
| θ° |  | 0.01 μM | 0.05 μM | 0.2 μM | 1 μM | 5 μM | 50 μM |
| 1 | 73 | 69 | 61 | 50 | 69 | 76 | 12 |
| 2 | 88 | 51 | 57 | 45 | 60 | 56 | 5 |
| 3 | 73 | 64 | 51 | 59 | 49 | 42 | 13 |
| 4 | 81 | 60 | 66 | 48 | 28 | 58 | 5 |
| 5 | 77 | 60 | 54 | 80 | 58 | 44 | 6 |
| 6 | 72 | 68 | 64 | 63 | 29 | 72 | 84 |
| 7 | - | 72 | - | 42 | - | - | 61 |
| average | 77 | 63 | 59 | 55 | 49 | 58 | 27 |
| SD | 6 | 7 | 6 | 13 | 17 | 14 | 32 |

**7.15 2m**

| elongation | Control | **2m** | | | | | |
| --- | --- | --- | --- | --- | --- | --- | --- |
| cm |  | 0.01 μM | 0.05 μM | 0.2 μM | 1 μM | 5 μM | 50 μM |
| 1 | 0.76 | 0.91 | - | 0.63 | 0.49 | 1.20 | 0.77 |
| 2 | 0.63 | 0.77 | 0.77 | 0.34 | - | 1.06 | 0.41 |
| 3 | 0.51 | 0.80 | 0.22 | 0.66 | - | 1.17 | 0.38 |
| 4 | 0.76 | 0.71 | 0.78 | 0.36 | 0.61 | 0.93 | 0.51 |
| 5 | 0.79 | 0.87 | 0.27 | 0.77 | 0.69 | 1.29 | 0.40 |
| 6 | 0.63 | 0.63 | - | 0.58 | 0.78 | 1.00 | 0.53 |
| 7 | 0.78 | 0.47 | - | 0.68 | 1.02 | 1.52 | 0.43 |
| average | 0.70 | 0.73 | 0.51 | 0.57 | 0.72 | 1.17 | 0.49 |
| SD | 0.11 | 0.15 | 0.31 | 0.17 | 0.20 | 0.20 | 0.13 |

| curvature | Control | **2m** | | | | | |
| --- | --- | --- | --- | --- | --- | --- | --- |
| θ° |  | 0.01 μM | 0.05 μM | 0.2 μM | 1 μM | 5 μM | 50 μM |
| 1 | 66 | 65 | - | 82 | 73 | 68 | 84 |
| 2 | 53 | 67 | 66 | 61 | - | 74 | 85 |
| 3 | 74 | 57 | 76 | 74 | - | 76 | 88 |
| 4 | 72 | 64 | 73 | 67 | 77 | 36 | 73 |
| 5 | 64 | 60 | 63 | 74 | 81 | 70 | 60 |
| 6 | 69 | 76 | - | 72 | 67 | 60 | 23 |
| 7 | 65 | 67 | - | 60 | 85 | 70 | 75 |
| average | 66 | 65 | 70 | 70 | 77 | 65 | 70 |
| SD | 7 | 6 | 6 | 8 | 7 | 14 | 23 |

**7.16 2n**

| elongation | Control | **2n** | | | | | |
| --- | --- | --- | --- | --- | --- | --- | --- |
| cm |  | 0.01 μM | 0.05 μM | 0.2 μM | 1 μM | 5 μM | 50 μM |
| 1 | 0.88 | 1.12 | 1.03 | 1.19 | 1.06 | 1.09 |  |
| 2 | 0.81 | 1.07 | 0.72 | 1.10 | 0.96 | 1.14 |  |
| 3 | 0.70 | 0.94 | 0.98 | 0.95 | 1.27 | 1.09 |  |
| 4 | 1.07 | 1.38 | 0.81 | 1.15 | 0.98 | 1.04 |  |
| 5 | 0.77 | 0.87 | 0.94 | 1.00 | 1.26 | 0.92 |  |
| 6 | 0.70 | 1.10 | 1.20 | 0.87 | 1.22 | 1.54 |  |
| 7 | 0.87 | 1.11 | 0.88 | 1.09 | 1.01 | 1.16 |  |
| average | 0.83 | 1.08 | 0.94 | 1.05 | 1.11 | 1.14 |  |
| SD | 0.13 | 0.16 | 0.16 | 0.11 | 0.14 | 0.19 |  |

| curvature | Control | **2n** | | | | | |
| --- | --- | --- | --- | --- | --- | --- | --- |
| θ° |  | 0.01 μM | 0.05 μM | 0.2 μM | 1 μM | 5 μM | 50 μM |
| 1 | 88 | 74 | 59 | 37 | -17 | -8 | curing |
| 2 | 89 | 87 | 64 | 19 | 10 | -12 |  |
| 3 | 74 | 60 | 73 | 34 | 42 | 32 |  |
| 4 | 95 | 71 | 82 | 30 | -14 | -15 |  |
| 5 | 82 | 87 | 56 | 29 | -22 | 16 |  |
| 6 | 89 | 85 | 66 | 20 | -15 | 31 |  |
| 7 | 81 | 87 | 83 | 23 | 9 | 32 |  |
| average | 85.43 | 78.71 | 69.00 | 27.43 | -1.00 | 10.86 | - |
| SD | 6.90 | 10.63 | 10.68 | 6.95 | 22.85 | 21.88 | - |

**7.17 2o**

| elongation | Control | **2o** | | | | | |
| --- | --- | --- | --- | --- | --- | --- | --- |
| cm |  | 0.01 μM | 0.05 μM | 0.2 μM | 1 μM | 5 μM | 50 μM |
| 1 | 1.04 | 0.61 | 0.48 | 0.44 | 1.01 | 0.93 | 0.29 |
| 2 | 0.60 | 0.43 | 0.49 | 0.00 | 0.94 | 1.15 | 0.31 |
| 3 | 0.81 | 0.67 | 0.59 | 0.00 | 0.94 | 0.86 | 0.27 |
| 4 | 0.93 | 0.38 | 0.49 | 0.70 | 0.97 | 0.83 | 0.37 |
| 5 | 0.59 | 0.64 | 0.44 | 0.66 | 0.96 | 0.81 | 0.13 |
| 6 | 0.87 | 0.58 | 0.54 | 0.57 | 1.03 | 1.23 | 0.33 |
| 7 | 0.74 | 0.39 | 0.72 | 0.00 | 0.87 | 0.84 | 0.54 |
| average | 0.80 | 0.53 | 0.54 | 0.34 | 0.96 | 0.95 | 0.32 |
| SD | 0.17 | 0.12 | 0.09 | 0.33 | 0.05 | 0.17 | 0.12 |

| curvature | Control | **2o** | | | | | |
| --- | --- | --- | --- | --- | --- | --- | --- |
| θ° |  | 0.01 μM | 0.05 μM | 0.2 μM | 1 μM | 5 μM | 50 μM |
| 1 | 50 | 74 | 41 | 38 | -14 | -6 | 113 |
| 2 | 42 | 52 | 45 | - | 40 | 48 | 13 |
| 3 | 48 | 61 | 60 | - | -8 | 1 | 3 |
| 4 | 48 | 56 | 44 | 31 | 29 | -27 | -15 |
| 5 | 43 | 65 | 35 | 20 | -8 | -27 | -30 |
| 6 | 50 | 63 | 47 | -10 | 11 | -11 | -12 |
| 7 | 47 | 66 | 75 | - | 21 | -25 | -59 |
| average | 47 | 62 | 50 | 20 | 10 | -7 | 2 |
| SD | 3 | 7 | 14 | 21 | 21 | 27 | 54 |

**7.18 2p**

| elongation | Control | **2p** | | | | | |
| --- | --- | --- | --- | --- | --- | --- | --- |
| cm |  | 0.01 μM | 0.05 μM | 0.2 μM | 1 μM | 5 μM | 50 μM |
| 1 | 0.76 | 0.18 | 0.76 | - | - | 0.94 | 0.35 |
| 2 | 0.63 | 0.29 | 0.58 | 0.80 | 0.79 | 1.18 | 1.05 |
| 3 | 0.51 | 0.39 | 0.77 | 0.92 | 0.98 | 0.82 | 0.61 |
| 4 | 0.76 | 0.39 | 0.44 | 0.92 | 0.50 | 0.75 | 1.26 |
| 5 | 0.79 | 0.73 | 0.69 | 1.02 | 0.78 | 0.91 | 1.01 |
| 6 | 0.63 | 0.45 | 0.38 | 1.13 | 0.81 | 0.78 | 0.95 |
| 7 | 0.78 | 0.32 | 0.84 | 0.76 | 0.91 | 0.80 | 0.86 |
| average | 0.70 | 0.39 | 0.64 | 0.93 | 0.79 | 0.88 | 0.87 |
| SD | 0.11 | 0.17 | 0.18 | 0.14 | 0.16 | 0.15 | 0.30 |

| curvature | Control | **2p** | | | | | |
| --- | --- | --- | --- | --- | --- | --- | --- |
| θ° |  | 0.01 μM | 0.05 μM | 0.2 μM | 1 μM | 5 μM | 50 μM |
| 1 | 66 | 67 | 49 | - | - | - | -22 |
| 2 | 53 | 63 | 57 | 31 | 11 | - | 85 |
| 3 | 74 | 53 | 53 | 20 | 3 | 5 | 17 |
| 4 | 72 | 60 | 40 | 51 | -73 | 5 | 54 |
| 5 | 64 | 67 | 60 | 8 | 3 | - | 52 |
| 6 | 69 | 63 | 56 | 29 | 9 | 5 | -20 |
| 7 | 65 | 68 | 55 | 14 | 3 | - | -29 |
| average | 66 | 63 | 53 | 26 | -7 | 5 | 20 |
| SD | 7 | 5 | 7 | 15 | 32 | 0 | 45 |

**[*Arabidopsis*]**

**7.19 (for Figure 4)**

|  | MOCK | | 10 nM | | 50 nM | | 100 nM | | 500 nM | | 1 μM | |
| --- | --- | --- | --- | --- | --- | --- | --- | --- | --- | --- | --- | --- |
|  | length | curvature | length | curvature | length | curvature | length | curvature | length | curvature | length | curvature |
|  | 6.60 | 78.1 | 7.11 | 126.3 | 7.38 | 79.7 | 4.79 | 273.8 | 5.26 | 149.5 | 6.27 | 125.1 |
|  | 6.49 | 89.3 | 6.75 | 109.7 | 6.27 | 55.6 | 5.98 | 49.8 | 6.29 | 0.0 | 4.79 | 219.8 |
|  | 5.99 | 86.8 | 6.71 | 85.9 | 4.75 | 107.7 | 7.02 | 85.3 | 3.58 | 349.7 | 6.16 | 267.0 |
|  | 5.02 | 92.4 | 6.63 | 90.0 | 6.89 | 63.4 | 5.22 | 84.8 | 4.44 | 240.3 | 6.33 | 307.1 |
|  | 5.72 | 101.0 | 6.75 | 125.0 | 4.91 | 94.6 | 4.01 | 69.1 | 4.21 | 180.0 | 5.84 | 255.1 |
|  | 5.02 | 74.4 | 4.88 | 76.6 | 6.38 | 110.3 | 5.35 | 32.0 | 4.46 | 180.0 | 5.92 | 140.7 |
|  | 6.78 | 96.0 | 7.72 | 129.8 | 5.26 | 98.0 | 6.26 | 71.6 | 7.39 | 86.4 | 4.99 | 93.8 |
|  | 7.21 | 77.2 | 7.40 | 79.1 | 5.79 | 12.3 | 6.47 | 84.0 | 5.17 | 294.7 | 2.83 | -251.6 |
|  | 6.08 | 86.7 | 5.64 | 121.8 | 5.68 | 138.4 | 6.26 | 70.0 | 7.71 | 141.7 | 5.32 | 309.8 |
|  | 7.27 | 97.4 | 6.57 | 57.7 | 5.08 | 67.6 | 6.22 | 56.3 | 5.80 | 52.9 | 4.14 | -253.3 |
|  | 4.50 | 75.3 | 5.86 | 110.6 | 7.07 | 136.0 | 6.88 | 85.9 | 7.85 | 165.0 | 3.96 | -242.3 |
|  | 9.20 | 87.4 | 3.91 | 133.0 | 7.02 | 69.9 | 3.93 | 99.9 | 6.59 | -229.1 | 4.18 | -238.0 |
|  | 8.06 | 87.6 | 7.31 | 120.4 | 7.88 | 51.0 | 7.00 | 34.7 | 5.72 | 54.5 | 7.04 | 243.4 |
|  | 6.56 | 81.8 | 8.15 | 87.3 | 7.64 | 74.1 | 7.53 | 92.3 | 8.40 | 47.3 | 5.65 | 187.6 |
|  | 6.80 | 78.1 | 7.15 | 116.6 | 7.94 | 109.2 | 6.24 | 115.3 | 8.68 | 301.6 | 6.64 | 81.9 |
|  | 5.61 | 90.0 | 8.27 | 60.0 | 5.28 | 99.0 | 7.18 | 104.0 | 7.01 | 29.1 | 5.99 | 163.7 |
|  | 6.61 | 90.0 | 5.86 | 107.7 | 4.83 | 95.7 | 4.04 | 57.1 | 7.36 | -230.5 | 6.54 | 208.3 |
|  | 5.77 | 93.2 | 7.61 | 62.6 | 6.49 | 114.8 | 5.18 | 68.7 | 6.00 | -202.6 | 3.39 | -180.0 |
|  | 6.44 | 80.5 | 7.68 | 61.7 | 4.89 | 108.4 | 5.52 | 79.4 | 4.96 | 48.6 | 5.43 | 4.9 |
|  | 8.05 | 78.4 | 7.21 | 137.3 | 5.48 | 84.6 | 6.96 | 155.4 | 5.92 | 168.1 | 6.40 | 168.9 |
|  | 7.21 | 91.5 | 7.07 | 54.7 | 7.87 | 98.1 | 6.57 | 90.0 | 5.30 | -107.0 | 5.78 | -353.7 |
|  | 5.74 | 114.2 | 4.09 | 98.2 | 6.82 | 88.3 | 3.62 | 95.2 | 3.05 | -220.6 | 6.24 | 357.3 |
|  | 6.31 | 99.5 | 4.47 | 61.4 | 3.61 | 67.5 | 5.03 | 118.8 | 5.63 | 265.2 | 3.78 | 0.0 |
|  | 4.85 | 97.5 | 7.47 | 80.0 | 7.53 | 92.2 | 5.00 | 110.6 | 5.40 | -265.2 | 4.03 | -48.6 |
|  | 4.07 | 86.2 | 6.11 | 68.3 | 6.04 | 74.5 | 5.03 | 239.5 | 8.41 | -219.8 | 7.18 | 137.3 |
|  | 6.65 | 107.2 | 5.72 | 123.5 | 8.26 | 85.2 | 5.46 | 58.0 | 7.61 | -119.7 | 6.24 | 126.5 |
|  | 8.28 | 109.3 | 6.38 | 137.7 | 8.39 | 63.4 | 5.16 | 212.0 | 7.35 | -180.0 | 5.04 | -223.2 |
|  | 8.11 | 72.0 | 7.05 | 113.2 | 6.51 | 118.4 | 6.58 | 148.0 | 7.66 | 270.0 | 6.95 | -121.7 |
|  | 9.43 | 67.5 | 7.84 | 87.0 | 8.22 | 93.6 | 6.85 | 162.6 | 5.43 | -218.1 | 8.24 | -6.4 |
|  | 6.43 | 75.3 | 6.11 | 44.4 | 7.66 | 91.8 | 6.91 | 58.4 | 7.29 | 80.7 | 8.77 | -199.9 |
|  | 7.36 | 83.8 | 5.32 | 58.0 | 8.95 | 88.1 | 7.90 | 127.7 | 7.74 | 235.8 | 7.45 | -65.6 |
|  | 6.83 | 100.7 | 7.86 | 124.3 | 8.26 | 100.5 | 5.73 | 102.8 | 6.27 | 107.4 | 4.82 | -157.3 |
|  | 8.34 | 103.0 | 6.14 | 106.9 | 10.42 | 80.2 | 6.99 | 107.1 | 8.76 | 273.8 | 3.09 | -232.1 |
|  | 5.48 | 96.1 | 6.06 | 115.3 | 6.26 | 75.8 | 6.78 | 0.0 | 8.25 | 351.6 | 3.43 | -194.1 |
|  | 6.26 | 97.1 | 6.92 | 96.6 | 4.95 | 137.0 | 4.46 | 136.3 | 6.99 | 266.6 | 6.60 | 164.1 |
|  | 7.19 | 93.1 | 6.07 | 113.6 | 7.57 | 77.5 | 6.73 | 72.9 | 7.60 | -310.3 | 5.77 | -67.3 |
|  | 7.89 | 90.0 | 7.16 | 76.0 | 6.81 | -22.4 | 6.06 | 112.8 | 4.82 | -155.1 | 5.28 | -204.0 |
|  | 6.49 | 101.3 | 3.77 | 140.5 | 8.47 | 45.0 | 7.37 | 120.5 | 7.44 | 24.0 | 7.00 | 232.3 |
|  | 8.03 | 92.5 | 6.62 | 76.4 | 6.39 | 46.9 | 5.64 | 137.7 | 5.51 | 251.6 | 7.24 | 233.1 |
|  | 6.57 | 80.5 | 5.30 | 116.6 | 4.15 | 99.5 | 5.07 | 138.0 | 4.56 | -259.7 | 6.77 | 231.3 |
|  | 7.67 | 91.7 | 7.23 | 98.7 | 7.27 | 125.8 | 6.45 | 54.5 | 5.37 | -80.0 | 6.19 | 276.7 |
|  | 5.58 | 71.5 | 6.50 | 77.5 | 7.73 | 71.6 | 6.71 | 63.4 | 5.57 | 79.5 | 5.65 | 209.9 |
|  | 7.34 | 90.0 | 6.80 | 102.3 | 5.63 | 26.6 | 6.76 | 52.7 | 6.21 | 143.7 | 6.52 | 237.3 |
|  | 7.87 | 75.1 | 5.97 | 23.5 | 7.33 | 107.7 | 7.50 | 133.3 | 4.23 | 180.0 | 7.10 | 114.9 |
|  | 7.41 | 89.1 | 6.28 | 56.6 | 6.34 | 191.8 | 7.06 | 0.0 | 6.53 | 155.2 | 5.94 | 59.0 |
|  | 3.95 | 90.0 | 7.82 | 98.7 | 6.71 | 137.5 | 6.23 | 234.2 | 6.10 | 242.1 | 7.91 | -181.3 |
|  | 6.83 | 81.7 | 4.83 | 68.9 | 8.25 | 125.5 | 8.78 | 310.2 | 4.16 | 231.9 | 6.07 | -219.3 |
|  | 7.67 | 71.6 | 6.50 | 31.5 | 8.39 | 159.1 | 6.62 | 131.6 | 6.95 | 288.4 | 5.67 | 148.2 |
|  | 7.47 | 84.8 | 6.19 | 90.0 | 6.98 | 46.6 | 5.49 | 87.4 | 5.98 | 75.1 |  |  |
|  | 6.86 | 91.8 | 4.94 | 94.6 |  |  | 6.73 | 128.2 | 6.99 | 254.1 |  |  |
|  | 5.78 | 74.2 | 6.14 | 101.7 |  |  | 4.97 | 270.0 |  |  |  |  |
|  | 5.73 | 93.3 |  |  |  |  | 3.39 | 211.0 |  |  |  |  |
|  | 6.97 | 84.4 |  |  |  |  | 6.02 | 45.0 |  |  |  |  |
|  |  |  |  |  |  |  | 4.02 | 58.4 |  |  |  |  |
| Avr | 6.69 | 88.1 | 6.43 | 92.9 | 6.75 | 89.0 | 5.99 | 109.2 | 6.24 | 69.4 | 5.80 | 38.9 |
| STDV | 1.18 | 10.4 | 1.09 | 28.8 | 1.39 | 37.0 | 1.16 | 66.5 | 1.41 | 192.3 | 1.34 | 199.4 |
|  | n=53 |  | n=51 |  | n=49 |  | n=54 |  | n=50 |  | n=48 |  |

| **9. Primers used in this study.** | |
| --- | --- |
| **name** | **sequence** |
| 18S rRNA-F | AAACGGCTACCACATCCAAG |
| 18S rRNA-R | CCTCCAATGGATCCTCGTTA |
| IAA5-F | TGAAGGAAAGTGAATGTGTACCAA |
| IAA5-R | GCACGATCCAAGGAACATTT |
| GH3.2-F | ACGTCACTTGTGGAGCTTGC |
| GH3.2-R | CCCGTGACACGAAGAATGTC |
| GUS-F | GTTCGCCGATGCAGATATTC |
| GUS-R | CAGCACGATACGCTGGCCTG |
|  |  |

**10. References**

1. Augustine, J. K., Bombrun, A., Venkatachaliah, S. & Jothi, A. Titanium mediated olefination of aldehydes with α-haloacetates: an exceptionally stereoselective and general approach to (Z)-α-haloacrylates. *Org. Biomol. Chem.* **11**, 8065 (2013).

2. Funabiki, K., Murata, E., Fukushima, Y., Matsui, M. & Shibata, K. Montmorillonite K 10 (clay) catalyzed hydrolysis of aryl-substituted α,β-difluoroallyl alcohols leading to (Z)-α-fluoro-β-aryl-substituted acrylaldehydes. *Tetrahedron* **55**, 4637–4642 (1999).

3. Shindo, M., Sato, Y. & Shishido, K. A highly stereoselective synthesis of tri- and tetrasubstituted olefins via ynolates. *Tetrahedron Lett.* **39**, 4857–4860 (1998).

4. Matsuda, T. & Sakurai, Y. Palladium-Catalyzed Ring-Opening Alkynylation of Cyclopropenones. *European J. Org. Chem.* **2013**, 4219–4222 (2013).

5. Du, Q., Neudörfl, J.-M. & Schmalz, H.-G. Chiral Phosphine-Phosphite Ligands in Asymmetric Gold Catalysis: Highly Enantioselective Synthesis of Furo[3,4- d ]-Tetrahydropyridazine Derivatives through [3+3]-Cycloaddition. *Chem. - A Eur. J.* **24**, 2379–2383 (2018).

6. Krafft, M. E., Vidhani, D. V., Cran, J. W. & Manoharan, M. Solvent controlled mechanistic dichotomy in a Au(iii)-catalyzed, heterocyclization triggered, Nazarov reaction. *Chem. Commun.* **47**, 6707 (2011).

7. Lautens, M., Maddess, M. L., Sauer, E. L. O. & Ouellet, S. G. Enantioselective Allylation of β , γ -Unsaturated Aldehydes Generated via Lewis Acid Induced Rearrangement of 2-Vinyloxiranes. *Org. Lett.* **4**, 83–86 (2002).

8. Ohno, H. *et al.* Double C-H Functionalization in Sequential Order: Direct Synthesis of Polycyclic Compounds by a Palladium-Catalyzed C-H Alkenylation-Arylation Cascade. *Chem. - A Eur. J.* **18**, 5352–5360 (2012).

9. Wrobel, J. *et al.* Syntheses of tolrestat analogs containing additional substituents in the ring and their evaluation as aldose reductase inhibitors. Identification of potent, orally active 2-fluoro derivatives. *J. Med. Chem.* **34**, 2504–2520 (1991).

10. Molander, G. A. & Fumagalli, T. Palladium(0)-Catalyzed Suzuki−Miyaura Cross-Coupling Reactions of Potassium Aryl- and Heteroaryltrifluoroborates with Alkenyl Bromides. *J. Org. Chem.* **71**, 5743–5747 (2006).

11. Mal, K., Sharma, A., Maulik, P. R. & Das, I. PPh3 HBr-DMSO Mediated Expedient Synthesis of γ-Substituted β,γ-Unsaturated α-Ketomethylthioesters and α-Bromo Enals: Application to the Synthesis of 2-Methylsulfanyl-3( 2 H )-furanones. *Chem. - A Eur. J.* **20**, 662–667 (2014).

12. He, T. *et al.* Platinum(IV)-Catalyzed Regioselective Synthesis of Highly Substituted 4 H -Cyclopenta[b]furans via Cascade Hetero- cyclization of 2-(1-Alkynyl)-3-aryl-2-propenals with Arylethenes. *Adv. Synth. Catal.* **355**, 365–369 (2013).
